# The Uptake and Metabolism of Amino Acids, and Their Unique Role in the Biology of Pathogenic Trypanosomatids

**DOI:** 10.3390/pathogens7020036

**Published:** 2018-04-01

**Authors:** Letícia Marchese, Janaina de Freitas Nascimento, Flávia Silva Damasceno, Frédéric Bringaud, Paul A. M. Michels, Ariel Mariano Silber

**Affiliations:** 1 Laboratory of Biochemistry of Tryps—LaBTryps, Department of Parasitology, Institute for Biomedical Sciences, University of Sao Paulo, Av. Lineu Prestes, 1374, São Paulo 05508-000, SP, Brazil; lets.marchese@gmail.com (L.M.); janaina_biomed@hotmail.com (J.d.F.N.); flaviadamasceno@usp.br (F.S.D.); 2 Laboratoire de Microbiologie Fondamentale et Pathogénicité (MFP), Université de Bordeaux, CNRS UMR-5234, 146, rue Léo Saignat, Zone Nord, Bâtiment 3A, 33076 Bordeaux, France; frederic.bringaud@u-bordeaux.fr; 3 Centre for Immunity, Infection and Evolution and Centre for Translational and Chemical Biology, School of Biological Sciences, Ashworth Laboratories, Charlotte Auerbach Road, The University of Edinburgh, Edinburgh EH9 3FL, UK; paul.michels@ed.ac.uk

**Keywords:** amino acid metabolism, amino acid uptake, bioenergetics, stress management, autophagy, host-parasite interaction

## Abstract

*Trypanosoma brucei*, as well as *Trypanosoma cruzi* and more than 20 species of the genus *Leishmania*, form a group of flagellated protists that threaten human health. These organisms are transmitted by insects that, together with mammals, are their natural hosts. This implies that during their life cycles each of them faces environments with different physical, chemical, biochemical, and biological characteristics. In this work we review how amino acids are obtained from such environments, how they are metabolized, and how they and some of their intermediate metabolites are used as a survival toolbox to cope with the different conditions in which these parasites should establish the infections in the insects and mammalian hosts.

## 1. Introduction

Trypanosomes are a group of flagellated protists living in a wide range of associations with other organisms, ranging from mutualism to parasitism. Inside this group we can find two genera which include species that are pathogenic to humans: *Trypanosoma* and *Leishmania*. The genus *Trypanosoma* groups two species causing human diseases: *Trypanosoma cruzi* and *Trypanosoma brucei*; in contrast, the genus *Leishmania* comprises more than twenty species affecting human health. All these pathogens have a complex life cycle involving several mammalian species and insects, which are responsible for the main mechanisms of transmission among mammals. Therefore, the life cycles of these parasites involve the establishment of the infection and survival inside different hosts. The different territories that these parasites should colonize inside each kind of host determine the need for a flexible and quickly adaptable metabolism to deal not only with their energy requirements, but also with a variety of stressing conditions predominant in the different environments in which these organisms live [1].

**Figure 1:**
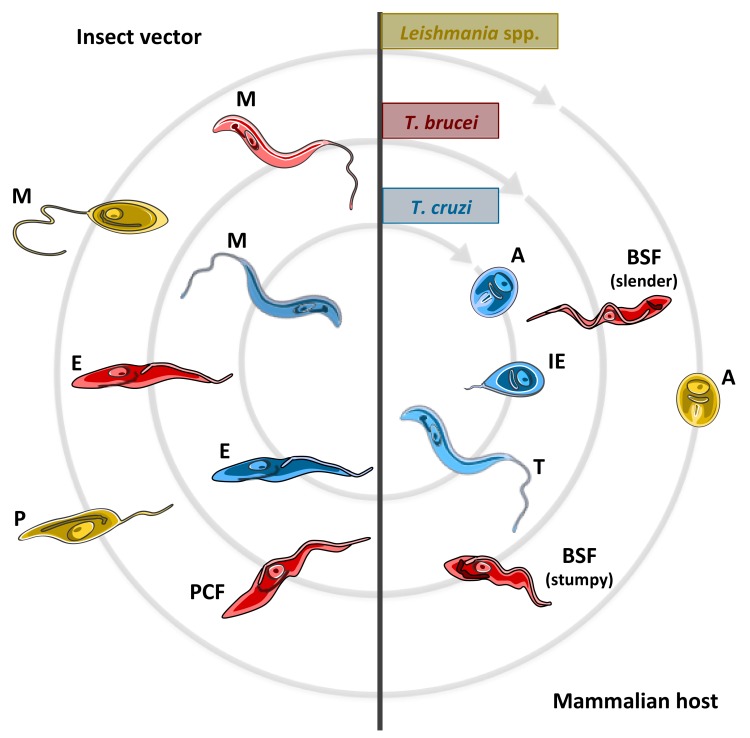
Life cycle of TriTryps parasites (*T. brucei*, *T. cruzi* and *Leishmania* spp.). The names of cell forms are abbreviated as A: amastigote, BSF: bloodstream trypomastigote, E: epimastigote, IE: intracellular epimastigote, M: metacyclic, P: promastigote, PCF: procyclic, and T: trypomastigote (adapted from [2]).

Briefly, the life cycles of *T. cruzi*, *T. brucei* and *Leishmania* spp. are schematized in Figure 1 and can be summarized as follows:

(a) *T. cruzi*, the causative agent of Chagas disease, infects the insect midgut after a bloodmeal on an infected mammal, having the non-replicative, infective trypomastigote forms circulating in its blood. In the triatomine gut, these trypomastigotes will differentiate into the replicative (non-infective for mammals) epimastigote forms, which are able to colonize the insect midgut. In the posterior region of the digestive tube, parasites differentiate into the infective, non-replicative metacyclic trypomastigotes [3]. During a next bloodmeal, the triatomine insect will defecate, expelling metacyclic trypomastigotes, which enter into contact with the skin or mucosa of the mammal being bitten. The parasites internalize into this new host through wounds in the skin or exposed mucosa, and must invade cells to reach the cytosol where they differentiate into replicative forms denominated amastigotes [4]. After several cycles of cell division, amastigotes differentiate into a transient replicative intracellular stage named intracellular epimastigotes [5], and, finally, these epimastigote-like forms differentiate into the infective trypomastigotes which burst from the infected cells into the extracellular environment. Trypomastigotes can have two fates, to infect neighbor cells or to reach the bloodstream. Once they reach the bloodstream they can infect other cells in neighboring or distant tissues, or they can be taken up by a new insect which will transmit them to other mammals [6].

(b) *T. brucei* is transmitted by the obligatory blood-feeding tsetse flies. The tsetse takes up bloodstream forms (BSF) of *T. brucei* during a bloodmeal on an infected mammal [7]. Two types of BSF can be distinguished on the basis of morphological, biochemical, and biological characteristics: the long-slender BSF, and the stumpy BSF. Long-slender BSF are replicative, and are responsible for the long-term parasitemia in the mammalian host. When long-slender BSF reach a certain population density, which is sensed through a secreted “stumpy induction factor” (SIF), they differentiate into the non-replicative stumpy forms [8]. These latter BSF forms are the only ones that are able to differentiate into the replicative procyclic forms (PCF) inside the insect and survive in the conditions present in the insect midgut [9]. PCF replicate and initiate their migration to the salivary gland. During this journey, they undergo differentiation into procyclic epimastigotes, a process that is completed once the salivary gland is reached [10]. Upon having colonized the salivary gland, they finally differentiate into the infective, non-dividing metacyclic forms, which will be inoculated in a new mammalian host during the next bloodmeal of the insect. Metacyclics will differentiate into long-slender BSF in order to replicate in the bloodstream to establish the infection. Eventually, BSF can cross the blood brain barrier and reach the central nervous system [11]. 

(c) *Leishmania* spp. are transmitted by female phlebotomine sandflies which become infected when they take their bloodmeal on an infected mammal [12]. During the bloodmeal, phlebotomines ingest infected macrophages, which are lysed by hydrolytic enzymes present in the insect digestive tube. The released amastigotes differentiate into proliferative promastigotes, which start to replicate in the midgut. Upon nutrient depletion and low pH, promastigotes differentiate into non-replicative, infective metacyclic promastigotes, which accumulate mostly in the stomodeal valve. During a next bloodmeal, these metacyclic forms will be inoculated into the mammalian host [13]. Components of the sandfly saliva can function as attractors for the host macrophages, which migrate to the region of the bite and phagocytize the *Leishmania* parasites [14]. Once inside the parasitophorous vacuole, metacyclic promastigotes can differentiate into replicative amastigotes. These amastigotes secrete a series of proteins that can modulate, or even subvert, the role of lysosomal-derived lytic enzymes, allowing the parasites to customize the vacuole as an adequate niche for replicating and establishing the infection [15]. The infection can spread to other phagocytic cells directly through the release of infective amastigotes from the macrophages. Additionally, other phagocytic host cells can become infected by the uptake of *Leishmania*-infected cells: intracellular amastigotes are able to induce in their host cells an apoptosis-like process causing their phagocytosis that allows the amastigotes to infect a new cell [15]. Cells infected with *Leishmania* spp. can be ingested by a (non-infected) sandfly, which will be able to transmit it to a new mammalian host.

As mentioned above, *T. brucei*, *T. cruzi*, and *Leishmania* spp. have insects and mammals as hosts. Their biology, the niches they colonize inside each host, and the mechanisms used against the host defenses have similarities and differences. For example, *T. brucei* is transiently present in the insect midgut: once they establish the infection they colonize the salivary gland. *Leishmania* spp. colonize the midgut and the proboscis of the insect, and is inoculated when the insect regurgitates during their bloodmeal. Differently, *T. cruzi* transit through the entire digestive tube, and eventually colonizes its entire epithelium, including the terminal portion, and the infected parasites are expelled with the faeces. In the mammalian host, the replicative stages of *Leishmania* spp. and *T. cruzi* are the amastigotes, which are strictly intracellular. However, the amastigotes of *Leishmania* spp. are intracellularly located in a parasitophorous vacuole in phagocytic cells (mostly macrophages), while *T. cruzi* (which is able to invade almost any nucleated cell) is transiently present in a parasitophorous vacuole, and then escapes to the host cell’s cytosol. Differently, *T. brucei* is strictly extracellular, and was classically assumed to being able to colonize the bloodstream and the central nervous system [11,16]. More recently, their ability to survive in the skin and the extracellular interstices of fat cells in adipose tissue was shown [17]. The indicated similarities and differences between these groups of organisms probably determined that each one evolved its metabolism to survive in the different environments they colonize. In this respect, amino acid metabolism is crucial for these parasites far beyond their conventional roles as sources of carbon and energy, and building blocks of proteins. In this review, we have compiled information about the relevance of amino acid transport and metabolism for involvement in critical biological processes during the life cycle of these parasites, such as the establishment of the infection within the mammalian and insect hosts, differentiation, modulation of the cell cycle and resistance to extreme stress conditions. 

A note on the nomenclature used: When genome sequencing of trypanosomatid organisms was initiated, one reference strain (or species, in the case of *Leishmania*) was chosen for each of three human diseases caused by trypanosomatids (sleeping sickness, Chagas disease, and leishmaniasis). The term TriTryps was then introduced to refer to *T. brucei*, *T. cruzi*, and *L. major* in most of comparative studies among these species. Subsequently, the term TriTryps has been adopted to refer to the entire range of human pathogenic trypanosomatids and will be used in this sense in this paper. In the case of amino acids, the conventional three-letter code is used to refer to the L-isomers from the beginning of the text without being specifically spelled in full at the first mention. All other abbreviations are used after first giving the full term followed by introducing the abbreviation between parentheses. Finally, to avoid confusion about names and identities of enzymes, we followed the IUBMB recommendations for biochemical texts: they are identified firstly by both their more common name and their EC number. In most of cases, an abbreviation is introduced to make the text more readable, and this abbreviation is used further. A complete list of all enzymes cited in the text, as well as their abbreviations and EC numbers, are found in Supplementary Table 1.

## 2. Amino Acid Uptake

### 2.1. Amino Acid Transporters and the Identification of Their Putative Genes in Trypanosomatids

Metabolite transport systems allow entry of all essential nutrients into the cells, initially to the cytosol and, subsequently, into the respective organelles. This is, in fact, the initial step of any metabolic pathway, allowing the incorporation of exogenous sources of carbon, nitrogen, sulfur, and phosphorus [18,19]. The substrates of transporters have been classified into eight categories: 1: inorganic solutes; 2: carbohydrates; 3: amino acids and derivatives; 4: bases and derivatives; 5: vitamins, cofactors, signaling molecules and their precursors; 6: drugs, dyes, sterols, and toxic substances; 7: macromolecules; and 8: miscellaneous compounds [19]. 

With the advent of genome sequencing of TriTryps it has been possible to identify an enormous variety of coding sequences for amino acid transporters [20]. These genes are spread throughout the chromosomes, in multiple loci. One of the first attempts to identify genes encoding amino acid transporters (AAT) in trypanosomatids focused on a family grouping of H^+^/amino acid symporters, the AAAP family (amino acid/auxin permease, TC 2.A.18). This family is part of the electrochemical potential-driven transporters and includes permeases transporting multiple or single amino acids [21,22]. Some transporters are encoded by single-copy genes, such as the *T. brucei* AAT11 and AAT12 (TbAAT11 and TbAAT12), others comprise gene families with multiple copies, such as TbAAT5 (six copies) and TbAAT7 (11 copies). This diversity is also reflected in the origin of the AAT genes. For example, AAT11, 12, 15, and 21 have been proposed to be orthologs present in *T. cruzi, T. brucei*, and *L. major,* derived from a common ancestor. Differently, *Lm*AAT1 seems to have originated by tandem duplication in *L. major*, whereas *Tc*AAT32/*Tc*AAT36 might have appeared by transpositive duplication in *T. cruzi* [22,23]. 

As will be discussed later in this review, various AAT/AAAP have been characterized and related to particular roles beyond uptake of amino acids as nutrients, such as osmotic control, differentiation and infection. However, a considerable number of members of both groups still remain uncharacterized. In this context, genetic tools have been used in attempts to identify the specific function of transporters in trypanosomatids, especially the ones related to drug resistance. For example, two independent RNAi library screens performed in BSF *T. brucei* indicated that the amino acid transporter TbAAT6 is most likely responsible for the uptake of DL-α-difluoromethylornithine (DFMO, also commercially known as eflornithine), because its knockdown conferred resistance against this drug [24,25,26]. The application of RNAi screens in trypanosomatids is limited to *T. brucei*, the only species of this family in which the RNAi machinery has been extensively used for reverse genetic approaches [27]. Predictably, the development of high-throughput CRISPR-Cas9 for use in other trypanosomatids will facilitate the discovery and characterization of novel amino acid transporters [28].

### 2.2. Biochemical Characterization of Amino Acid Transporters

Amino acids are available in both mammalian and insect hosts of TriTryps at different concentrations and these metabolites can be consumed as energy source, what occurs mainly when they colonize insect vectors [29,30,31]. Amino acid transporters with a broad range of affinities and specificities have been described for TriTryps, probably reflecting the broad range of concentrations in which amino acids are present in the different hosts. In this review, we will approach the biochemically-described transport systems according to the representative groups of TriTryps.

#### 2.2.1. *Trypanosoma* *brucei*

Early studies of amino acid transport showed that *T. b. gambiense* BSF take up different amino acids, such as Ala, Arg, Asp, Glu, Gly, Leu, Lys, Met, Phe, Ser, Thr, and the non-proteinogenic amino acid ornithine (Orn). In these studies, the authors showed that, in most cases, such uptake was mediated by a membrane-associated transport system, but these systems were not fully characterized [32,33,34]. Initially, it was suggested that, based on their specificities, the transport systems formed discrete sets (collectively named *loci*), each one able to take up different amino acids. Five different loci were described: locus A transports Lys and, by binding Glu and Arg, stimulate Lys uptake; locus B transports Thr, Gly, and Ala; locus C transports Glu, this transport being inhibited by the binding of Phe, Met, or Thr; locus D transports Met and Phe; and locus E transports Arg and Lys, this transport being inhibited by its binding of Met and Phe [34]. Later, the Thr transport in *T. brucei* BSF was assessed at Thr concentrations close to those of the rat’s plasma, indicating a single transporter instead of a locus [35]. Almost simultaneously, Cys transport was described in the context of the design of a medium for axenic cultivation of BSF. Duszenko and collaborators [36] showed that these parasites efficiently incorporated Cys, but not cystine (oxidized Cys form).

It has been shown that Pro is a main carbon source for PCF grown in glucose-depleted conditions [37,38,39]. In the hemolymph of tsetse flies (*Glossina* species) Pro can reach concentrations as high as 60 mM, which was assumed to be used by *T. brucei* parasites during the infection [2,40]. This reinforced the interest in the characterization of Pro uptake in PCF of *T. b. brucei*. The kinetic characterization of Pro uptake pointed to a single high affinity active transporter (*K*_M_ 0.02 mM) inhibited by Ala and Cys [41]. It was also shown that the *V*_max_ value in parasites cultivated in glucose-depleted SDM80 medium was 2.6 times higher than that in parasites grown in glucose-rich SDM79 medium, indicating that Pro transport is down-regulated in the presence of glucose [38]. More recently, the RNAi library screening approach allowed the identification of the transporter responsible for uptake of the drug DFMO, which is TbAAT6, a low affinity transporter of neutral amino acids, including Pro, Gly, and Trp [42]. It is noteworthy that the biochemical characteristics (affinity and substrate specificity) reported for TbAAT6 expressed in *Xenopus laevis* oocytes and the PCF Pro transport system are markedly different [38,41,42]. In addition, TbAAT6 knockdown did not affect the growth of PCF in glucose-depleted conditions and Pro uptake rates, but it resulted in reduced sensitivity to DFMO [42]. This supports the view that *T. brucei*, like *T. cruzi* and *Leishmania* spp. (see below), expresses high-affinity and low-affinity Pro transport systems, the former being probably responsible for Pro metabolism in glucose-depleted conditions and at low Pro concentrations, and the latter (TbAAT6) being responsible for DFMO uptake.

Met transport systems have also been characterized in *T. b. brucei* [43,44]. Met uptake in both BSF and PCF of *T. b. brucei* occurs by a relatively high affinity (K_M_ 0.03 mM), proton-motive force dependent system and is inhibited by Leu, Phe, and Trp. However, in BSF Met, uptake showed a broader inhibition profile: D-Met, Leu, and Gln also inhibited Met uptake efficiently. These data suggest that different transporters are operative in the two stages [44]. Interestingly, low-affinity Met transporters with similar kinetic properties (K_M_ > 1 mM) have been described for *T. b. brucei* and *T. b. rhodesiense* BSF [43].

More recently, other members of the AAT family have been characterized. TbAAT5-3 and TbAAT16-1 have been heterologously expressed, showing high affinity for Arg and Lys (in both cases *K*_M_ in the low µM range) and high selectivity (including stereoselectivity, the D-isomer does not affect the transport). Peculiarly, down-regulation of these transporters affected BSF growth, demonstrating they are essential for the parasites [45]. In addition, it has been shown that *T. brucei* has two high-affinity Orn transporters, TbAAT10-1 and TbAAT2-4. TbAAT10-1 is selective for Orn, whereas TbAAT2-4 transports both Orn and His, which is consistent with inhibition of TbAAT2-4-mediated Orn uptake in the presence of high extracellular His levels. Orn uptake by both transporters is important for growth of BSF *T. brucei*, probably due to their role in providing Orn for polyamine biosynthesis [46].

#### 2.2.2. *Trypanosoma* *cruzi*

The study of amino acid uptake in *T. cruzi* was initiated with the demonstration that Arg and Lys uptake was mediated by transport systems [47,48,49]. Lys transport was described as being performed by three different systems, based on their sensitivity to neutral amino acids, with one of them being inhibited by Phe and Tyr, another by Pro, Ala, Met, and Cys, and the last one by Gly and branched-chain amino acids (BCAA), respectively [47]. Four of these amino acids that inhibit Lys transport (Gly, Val, Met, and Ala) have also been shown to be inhibitors of Arg transport [48]. The existence of these transport systems was also confirmed by Goldberg in *T. cruzi* epimastigotes [49], who proposed that Met is an inhibitor of both transporters, and Lys is an inhibitor of Arg uptake and vice versa. More recently, two Arg transport systems were fully characterized, a high-affinity transporter specific for Arg and a low-affinity transporter (*K*_M_ value for Arg 70-fold higher than that of the high-affinity system), which is inhibited by Met and, to a lesser extent, by Gly, Lys, and Tyr [50,51]. Later, a gene encoding a cationic amino-acid transporter, *Tc*AAP7, was identified in *T. cruzi*. It was biochemically characterized as a Lys permease through heterologous expression and through overexpression in epimastigotes. No kinetic parameters were reported for the recombinantly expressed *Tc*AAP7 protein, but the transport activity in epimastigotes showed a *K*_M_ of 23 µM. Interestingly, when epimastigotes were subjected to starvation in PBS supplemented with either glucose or Pro, Lys uptake was increased, whereas in Lys-supplemented PBS Lys uptake was considerably decreased. These data indicate the existence of an energy-dependent, substrate-sensing mechanism that regulates Lys transport [52].

A gene encoding an Arg permease (*Tc*AAAP411/*Tc*AAP3) was identified, and the biochemical characterization of the protein showed an activity with kinetic parameters compatible with the high-affinity Arg transporter previously characterized by Pereira and coworkers [53]. The influence of *Tc*AAP3 in Arg metabolism was also studied, showing that the intracellular Arg concentration increases and arginine kinase is significantly down-regulated in parasites that overexpressed *Tc*AAP3 [54]. Recently, a novel transporter (*Tc*CAT1.1) has been characterized, which was able to transport both Arg and Orn, although it showed high affinity for Arg and low affinity for Orn [55].

Pro uptake was also investigated in *T. cruzi*. The kinetic analysis indicated the presence of two relatively low-affinity active transport systems (named systems A and B) with different affinities. System A is H^+^ dependent, whereas system B is presumably dependent on ATP hydrolysis. System A is inhibited by Trp, Gly, Leu, Met, Cys, and Ala, while Cys, Ala, and Val inhibit system B [56]. A low affinity Pro carrier (*Tc*AAP24, the *Tb*AAT6 ortholog), transporting both the L- and D-Pro enantiomers, has more recently been characterized [57]. This unusual lack of stereospecificity for Pro can be related to the reported expression of a Pro racemase in *T. cruzi* and *Trypanosoma vivax* (but not in *T. brucei* and *Leishmania* spp.) [58].

The transport of two anionic amino acids, Asp and Glu, in *T. cruzi* was described to be mediated by at least two different systems with maximal activities at lower pH [59,60]. Asp uptake occurs with the same high affinity and specificity (only inhibited by Glu) in the epimastigote and trypomastigote forms. Glu transport, which was shown to be H^+^ dependent, occurs with 10-times less affinity than Asp, and was inhibited by Asp, Ala, Gln, Asn, and Met [60]. Notably, the rate of Asp uptake in epimastigotes increases upon nutrient starvation of the parasites [59], while such starvation had no effect on Glu uptake [60]. 

The transport of His and Gln, two amino acids metabolically related to Glu, was also characterized. His uptake is performed by a medium-affinity, highly specific, active system [61]. In contrast, the highly-specific Gln transporter can be considered of low affinity, with a *K*_M_ value in the mM range (similar to that of system A for Pro transport). Like the Glu transport system, both His and Gln uptake are H^+^ dependent and do not compete with other amino acids [Damasceno, F.S., Barisón, M.J., et al., submitted]. The transport of γ-aminobutyric acid (GABA), another Glu-related metabolite, showed kinetic parameters close to those of Glu uptake, but involves a different mechanism. GABA uptake is the only Na^+^-dependent amino acid transport characterized so far [60,62].

Cys uptake is mediated by a high-affinity system, showing an increased *V*_max_ under nutrient starvation and in stationary growth phase. Cys uptake seemed to be highly specific, since none of the amino acids tested (Pro, Ser, Gly, Met, Arg, Glu) significantly affected its transport rate [63]. 

The uptake of all BCAA (Val, Ile, and Leu) was shown to be performed by the same transport system, with relevant differences in affinity (*K*_M_^IIe^ 470 µM, *K*_M_^Leu^ 940 µM, *K*_M_^Val^ 1960 µM) and velocities. The system is H^+^ dependent and highly specific, since no other amino acids than the branched ones were able to decrease their incorporation [64].

#### 2.2.3. *Leishmania* spp.

The transport of Met was the first one studied in *Leishmania* spp. by Mukkada and Simon [65]. In promastigotes of *L. tropica*, this uptake occurs through a single system, following Michaelis-Menten kinetics. After 10 min of Met uptake, only 20% of this amino acid is metabolized, mainly to Met sulfoxide and cystathionine [65]. In addition, D-Met and some neutral amino acids (Ala and BCAA), as well as internal pools of Met, Lys, and Tyr, were able to inhibit Met transport [66]. Mukkada and collaborators [67] were also pioneers in the characterization of Pro transport in *Leishmania,* describing it as an active system with broad specificity, and inhibited by Ala, Met, and Val. Later, Bonay and Cohén [68] proposed the existence of two Na^+^-independent neutral amino-acid (including Met) transporters in *Leishmania*, one being more specific for Ala and Pro uptake and the other more specific for Phe and Leu uptake. The energy driving Pro transport in the *L. donovani* promastigotes was determined as being a proton-motive force comprising a transmembrane pH gradient and electrical potential [69]. Later, it was established that although the uptake of Pro has an optimal pH in the neutral region (7.0–7.5), its dependence on the electrochemical potential is constant over the entire pH range tested [70]. The pH dependence of Pro transport in *L. donovani* was also seen in intracellular forms. Different from promastigotes, in which uptake is inhibited at an acidic pH, that in amastigotes has an optimum at pH 5.5. Nonetheless, as in promastigotes, Pro transport is driven by the proton-motive force [71]. Another study showed that the uptake of Pro can occur by passive transport when this amino acid is the growth rate-limiting carbon and energy source in glucose-free conditions [72]. It is also regulated by the extracellular pH [71], with different kinetics compared to that operating in promastigotes grown and assayed at pH 7.0 [73]. The differences found in the transport of Pro between *L. donovani* life-cycle stages suggest that Pro can be captured by more than one system. This was confirmed by the identification of three different Pro transport systems: a cation-dependent (system A) and a cation-independent (system B) transporter in promastigotes, and a single cation-independent transporter in amastigotes (system C) [74]. The A and C systems have an optimum pH in the acidic range (pH 6.0 and 5.0, respectively); they are less specific for Pro and able to mediate uptake of several other amino acids. In contrast, system B is less sensitive to the extracellular pH and is more specific for Pro, while only Ala, Cys, Gln, and Gly inhibit it more than 50% [74]. 

Also biochemically characterized in *L. donovani* promastigotes is an Arg transporter that showed a high affinity and specificity, a Na^+^ dependence and pH sensitivity with an optimum pH at 7.5 [75]. Two more amino-acid transport systems were characterized in *L. amazonensis*. First, a single Glu transport system was found in promastigotes, which was shown to be energy dependent, sensitive to the extracellular H^+^ concentration (pH optimum at 6.0) and insensitive to K^+^ and Na^+^ and susceptible to inhibition by Gln, Asp, Asn, Ala, and Met [76]. Ser uptake was also characterized in *L. amazonensis* promastigotes and amastigotes. Its kinetic analysis showed a single transport system with similar characteristics in both forms, i.e., a medium affinity and pH responsiveness (pH optimum at 7.5). Similarly to what was observed for the Glu transport system, it was not affected by K^+^ or Na^+^, and was inhibited by other amino acids, in this case, Ala, Cys, Gly, Thr, and Val [77].

Some genes annotated as putatively-encoding amino-acid transporters were functionally characterized, allowing to provide a molecular identity to some of the biochemically-characterized systems. A neutral amino-acid transporter (LbAAP24), which was shown to transport mainly Pro and Ala, was identified and characterized by heterologous expression. It appeared to be a transporter with low affinity and high specificity for Pro and with an optimum pH at 6.5, identifying it as a promastigote-specific transport system A. Interestingly, LbAAP24 plays a critical role in amino-acid homeostasis and in hypotonic stress resistance [78]. Subsequently, an active and highly-specific, high-affinity Arg transporter (LdAAP3) was identified and characterized, with kinetic parameters similar to those of the system described by Kandpal et al. [79]. In addition, like for LdAAP24, a role was shown for LdAAP3 in maintaining Arg homeostasis in promastigotes [80]. This regulation was also reported in *L. amazonensis* [81,82].

Finally, transporter LdAAP7 was identified and characterized as a Lys-specific permease in *L. donovani*. Biochemical analysis of LdAAP7, heterologously expressed and in promastigotes, revealed similar kinetic parameters for Lys uptake (*K*_M_ 7 µM and 3 µM, respectively), however, different pH dependencies (pH optimum at 4.5 and 6.5, respectively) were found. In both cases, Lys uptake was highly specific and seemed to be regulated by the intracellular Lys pool. Its overexpression in promastigotes did not increase Lys transport. Furthermore, no uptake of Lys was observed in stationary phase promastigotes, in agreement with the notion that, until now, Lys seems not to be involved in other processes than the synthesis of proteins [52].

All amino acid transporters and systems that have been characterized in TriTryps are summarized in Table 1. 

## 3. Amino Acid Metabolism in TriTryps

### 3.1. Proline 

**Figure 2:**
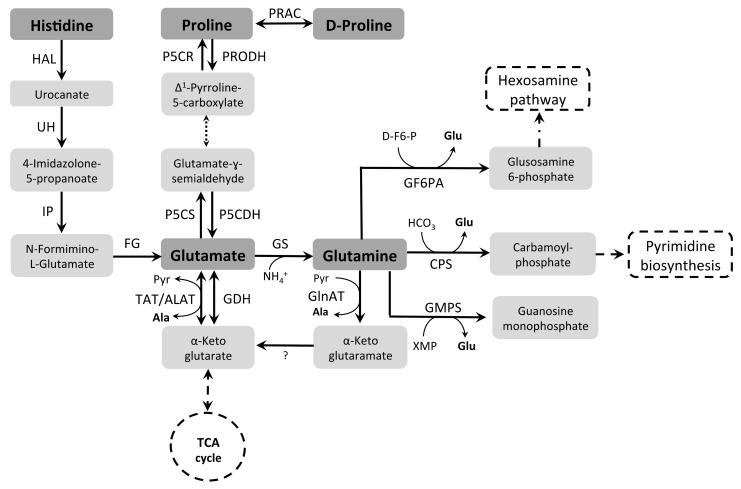
Metabolism of histidine, proline, glutamate, and glutamine. Enzymes: HAL: histidine ammonia lyase (EC 4.3.1.3); UH: urocanate hydratase (EC 4.2.1.49); IP: imidazolone propionase (EC 3.5.2.7); FG: formimidoylglutamase (EC 3.5.3.8); PRAC: proline racemase (EC 5.1.1.4); P5CR: pyrroline-5-carboxylate reductase (EC 1.5.1.2); P5CS: pyrroline-5-carboxylate synthase (EC not assigned); PRODH: proline dehydrogenase (EC 1.5.99.8); P5CDH: pyrroline-5-carboxylate dehydrogenase (EC 1.5.1.12); GDH: glutamate dehydrogenase (EC 1.4.1.3); GS: glutamine synthetase (EC 6.3.1.2); GF6PA: glutamine fructose-6-phosphate aminotransferase (EC 2.6.1.16); CPS: carbamoyl-phosphate synthase (EC 6.3.3.5); GMPS: GMP synthase (EC 6.3.5.2); ALAT: alanine aminotransferase (EC 2.6.1.2); TAT: tyrosine aminotransferase (EC 2.6.1.5); GlnAT: glutamine aminotransferase (EC 2.6.1.15). Metabolites: Pyr: pyruvate, Ala: L-alanine, XMP: xanthosine monophosphate, Glu: L-glutamate, D-F6-P: D-fructose 6-phosphate. The question mark (?) indicates unknown step.

Pro can be catabolized by enzymes of the tricarboxylic acid (TCA) cycle after its conversion into Glu through two enzymatic steps [2,38,92,93,94,95,96]. Pro catabolism was characterized in some detail in *T. cruzi* and *T. brucei*. It initiates via Pro dehydrogenase (PRODH, EC 1.5.99.8), which produces pyrroline-5-carboxylate (P5C) using FAD as an electron acceptor. As previously shown for other systems [97,98], PRODH-derived FADH_2_ is able to transfer electrons to the ubiquinone pool in the electron transport chain of trypanosomes [95]. In aqueous medium, P5C is converted through a non-enzymatic reaction to its open form glutamate-γ-semialdehyde (GSA) which is oxidized to Glu by a P5C dehydrogenase (P5CDH, EC 1.5.1.12) with a concomitant reduction of NAD(P)^+^ [96,99,100] (Figure 2).

Among the TriTryps, *T. brucei* was the first organism in which the first Pro oxidation step was characterized. As described for Pro uptake, the activity of TbPRODH in PCF is downregulated by the presence of glucose. Accordingly, in PRODH-deficient cell lines, the absence of glucose is lethal. Even more, it was shown that Pro is the only amino acid able to sustain the growth of PCF in a medium without glucose. In addition, the absence of glucose induces a 1000-fold increase of oligomycin sensitivity, which is explained by the oxidative phosphorylation dependency for ATP production when Pro is the major carbon source consumed by PCF [38]. Later, Mantilla et al. [39] showed that the second step of Pro catabolism is also essential for PCF in glucose-depleted conditions. Although P5CDH deficiency was not lethal in glucose-rich medium, TbP5CDH knockdown cells showed a slower growth rate than parental cells and a diminished mitochondrial membrane potential and capacity for ATP synthesis when cultured in medium without glucose. Remarkably, it was shown that the integrity of the Pro–Glu pathway is important for the PCF to survive in the tsetse fly midgut, a necessary condition for establishing infection in the tsetse vector.

The Pro–Glu pathway was also addressed in *T. cruzi*. It was shown that *Tc*PRODH is a FAD-dependent Pro oxidoreductase, which is able to reduce cytochrome c in an antimycin-sensitive manner [95]. This enzyme is up-regulated in intracellular epimastigotes, consistent with previous reports on its importance for differentiation of these forms to trypomastigotes [101]. P5CDH in *T. cruzi* was identified and characterized as a component of the inner mitochondrial membrane. It is able to reduce both NAD^+^ and NADP^+^, although the enzyme has a higher affinity for NAD^+^. *Tc*P5CDH is up-regulated in the infective stages (trypomastigotes and metacyclic trypomastigotes) and sustains energetically *T. cruzi* growth and the process of infecting host cells [96]. In contrast, the characterization of Pro catabolism in *Leishmania* spp. has not been performed yet, although two of its steps are known to also exist in *Leishmania* metabolism through genome data and by ancient and recent metabolic studies [2,30,92,102,103].

In addition to taking it up, most of organisms can synthesize Pro *de novo*. The most common biosynthesis pathway occurs from Glu through its phosphorylation to γ-glutamyl phosphate by γ-glutamyl kinase (γGK, EC 2.7.2.11), transferring a phosphoryl group from ATP to Glu. Then, γ-glutamyl phosphate is reduced to GSA by γ-glutamyl phosphate reductase (GPR, EC 1.2.1.41), with the simultaneous oxidation of NADPH [104,105]. In most higher eukaryotes, γGK and GPR activities are provided by the bifunctional enzyme P5C synthase [106,107] (P5CS, EC not assigned) and its product, GSA, is non-enzymatically converted into P5C, which serves as a substrate for P5C reductase (P5CR, EC 1.5.1.2) to produce Pro using NADPH or NADH as electron donors [108,109,110,111]. 

Alternatively, Pro might also be produced from Orn, connecting its metabolism to Arg metabolism through Orn aminotransferase (OAT, EC 2.6.1.13), which can provide GSA and Glu by transferring the amino group of Orn to α-ketoglutarate (α-KG) in a pyridoxal-5-phosphate-dependent reversible reaction [112]. However, there is as yet no biochemical evidence for the OAT enzyme in trypanosomatids, nor has a putative gene encoding such an enzyme been detected [20], and the urea cycle is not operative in these protists [102,113].

*T. brucei* is auxotrophic for Pro [39], whereas *T. cruzi* is capable of producing Pro in its cytosol from Glu via P5CS and P5CR. Interestingly, this Pro production in *T. cruzi* is NADPH-dependent and is regulated by the cytosolic pools of this cofactor (Marchese et al., unpublished data). Little is known about Pro biosynthesis in *Leishmania* spp., but it should be remarked that their genomes contain candidate genes for P5CS and P5CR [2,102]. In addition, Pro was not essential for growth of promastigotes in NM defined medium, strongly indicating that the biosynthetic pathway for this amino acid can be operative in these *Leishmania* [85]. 

Like some bacteria, the trypanosome species *T. cruzi* and *T. vivax* can interconvert L- and D-Pro through a Pro racemase (PRAC, EC 5.1.1.4) [58,114]. The first eukaryotic PRAC was identified as secreted in the culture medium by *T. cruzi* [115]. The *T. cruzi* genome encodes two isoforms of *Tc*PRACs with different kinetic properties, one secreted, and one remaining intracellularly [116,117]. The role of D-Pro, and the interconversion between L- and D-Pro by the *Tc*PRAC have not yet been completely elucidated, although there are some clues about the role of the enzyme in different aspects of the biology of *T. cruzi*, as will be discussed later in this review.

### 3.2. Glutamine, Histidine, and Glutamate

Glu can be considered a metabolic hub, which can be produced from a variety of metabolic sources (such as His, Gln, Pro, or the TCA cycle intermediate α-KG). In addition, it can be a precursor for these metabolites in most cells. Once Glu is available inside the cells, it can be reversibly converted into α-KG by the enzyme Glu dehydrogenase (GDH, EC 1.4.1.3) [118,119], transferring the amino group to H_2_O (forming NH_4_^+^), thus participating in energy metabolism [93,120]. Moreover, α-KG can be converted into malate, and further into pyruvate through the malic enzymes [121]. Alternatively, the amino group can be removed from Glu to be transferred to pyruvate by an Ala aminotransferase (ALAT, EC 2.6.1.2) or a Tyr aminotransferase (TAT, EC. 2.6.1.5) generating Ala and α-KG, respectively [122]. Remarkably, α-KG can be aminated to produce Glu, a mechanism to detoxify the excess of NH_4_^+^ produced by amino acid consumption. As mentioned, the amino group can be transferred to pyruvate or other keto-acids serving as substrates for the ALAT or TAT, to allow the recovery of α-KG, critical for keeping the TCA cycle working (Figure 2). 

Glu is also produced through the catabolism of other amino acids, such as Pro (as described above), His, and Gln. His can be converted into Glu through a four-enzymatic-steps degradation pathway [61]. The first step of His catabolism involves the formation of urocanate and NH_4_^+^ catalyzed by His ammonia lyase (HAL, EC 4.3.1.3). Next, urocanate hydratase (UH, EC 4.2.49) converts urocanate in 4-imidazolone-5-propianate (IPA) that is hydrolyzed by imidazolone propionase (IP, EC 3.5.2.7) resulting in N-formimine-L-glutamate. The last step involves the formation of Glu and formamide through a formimidoylglutamase (FG, EC 3.5.3.8) (Figure 2). 

According to the TriTryp genome database, *T. cruzi* is the only trypanosomatid containing genes for the four-step pathway converting His into Glu. This is in agreement with the capacity of *T. cruzi* to completely oxidize His, forming CO_2_ [61]. Transcriptomic data have shown that the first two enzymes of the His degradation pathway are primarily expressed in the invertebrate host stages [123]. Accordingly, an increase of the levels of urocanate, the product of the first step of His degradation, has been reported for stationary phase epimastigotes [124]. 

The availability of Gln depends on its uptake from the culture medium (Damasceno et al., unpublished data) and its biosynthesis catalyzed by Gln synthetase (GS, EC 6.3.1.2), which uses Glu and NH_4_^+^ as precursors, in a reaction that requires ATP. Incidentally, GS activity participates in the NH_4_^+^ detoxification and, probably through the resulting control of the low pH, in the parasite exit from the parasitophorous vacuole inside the host [125]. 

Gln can participate as nitrogen donor in the hexosamine pathway by the enzyme Gln fructose-6-phosphate aminotransferase (GF6PA, EC 2.6.1.16) (our unpublished data), as well as in the pyrimidine biosynthesis by the enzyme carbamoyl-phosphate synthase [126,127] (CPS EC 6.3.3.5) and in the GMP synthesis through the action of the enzyme GMP synthase [128] (GMPS, EC 6.3.5.2). Moreover, Gln is a substrate for the enzyme Gln aminotransferase (GlnAT, EC 2.6.1.15) that transfers the amino group from Gln to pyruvate, generating Ala and α-ketoglutaramate [129].

*Leishmania* can take Glu up from the culture medium [76] and oxidize it intracellularly by the TCA cycle, thus contributing to the energy metabolism [102]. Additionally, in *Leishmania* spp., Glu can be converted into Gln by a GS [130], as occurs in *T. cruzi*, using Glu and NH_4_^+^ as substrates. Moreover, the importance of the enzyme GF6PA (which uses Gln as substrate, and participates in the hexosamine pathway) was demonstrated. Promastigote GF6PA null mutants are unable to proliferate inside macrophages [131].

### 3.3. Arginine and Ornithine

Arg forms, in most eukaryotes, one of the central metabolic nodes, connecting the urea cycle with Glu/Pro metabolism through its conversion to Orn by an arginase (ARG, EC 3.5.3.1) and then to GSA/P5C by OAT. As explained above, the oxidation of P5C by P5CDH yields Glu, which connects with the TCA cycle through α-KG. This makes it crucial for cell viability since it is a precursor of Orn, urea, NO, polyamines (see below), and other amino acids, such as Pro [132]. When arginase is present, a major metabolic fate of Arg is the synthesis of polyamines. It happens through a complex pathway that involves four steps. First, Arg is converted into Orn through the action of an arginase (ARG, EC 3.5.3.1). Orn is then decarboxylated in a reaction catalyzed by Orn decarboxylase (ODC, EC 4.1.1.17), generating putrescine. Next, spermidine synthase (SPDS, EC 2.5.1.16) adds an aminopropyl group to putrescine, finally generating spermidine. The last step uses a decarboxylated S-adenosylmethionine as an aminopropyl donor, which is an outcome of the action of the enzyme S-adenosylmethionine decarboxylase (AdoMetDC, EC 4.1.1.50). In mammals, there is a fifth step in which spermidine is converted to spermine [133]. 

**Figure 3:**
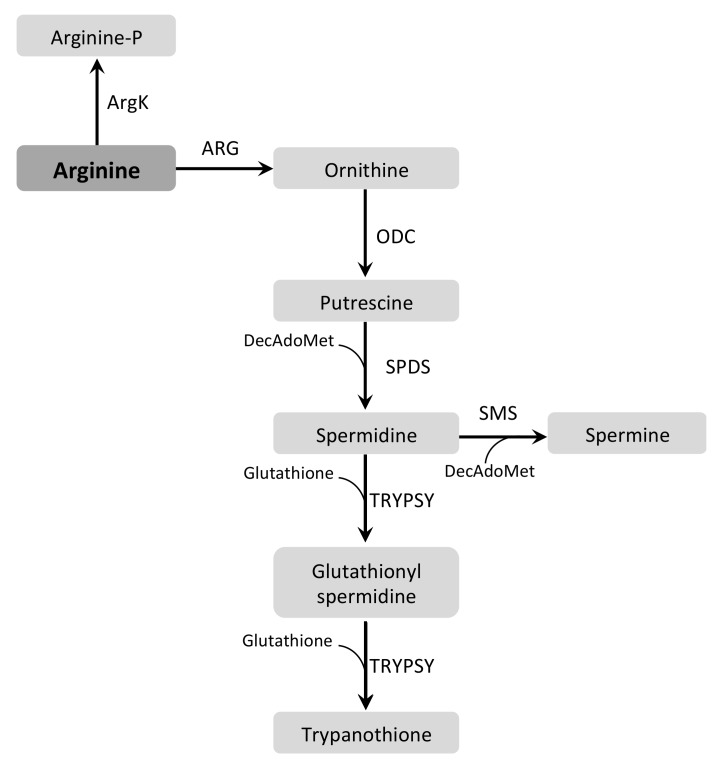
Metabolism of arginine and ornithine. **Enzymes:** ArgK: arginine kinase (EC 2.7.3.3); ARG: arginase (EC 3.5.3.1); ODC: ornithine decarboxylase (EC 4.1.1.17); SPDS: spermidine synthase (EC 2.5.1.16); SMS: spermine synthase (EC 2.5.1.2); TRYPSY: trypanothione synthase (EC 6.3.1.9). **Metabolite:** DecAdoMet: decarboxylated S-adenosyl methionine.

Interestingly, in *T. brucei* and *Leishmania* spp. this latter step is not present, while *T. cruzi*, in turn, seems to have lost the ODC coding sequence and is auxotroph for diamines [134]. In trypanosomatids, spermidine can be used to synthesize the redox agent trypanothione, which is specific to these organisms [135]. This synthesis occurs through a two-step pathway mediated by a single enzyme, the trypanothione synthetase (TRYPSY, EC 6.3.1.9) [136,137,138]. Trypanothione, in turn, can be reduced by the homolog of glutathione reductase, the trypanothione reductase (TRYPRD, EC 1.8.1.12) [139,140] (Figure 3). 

In *Leishmania* spp. the catabolism of Arg involves its conversion to Orn and urea, catalyzed by ARG. *Lm*ARG is compartmentalized in peroxisome-related organelles, named glycosomes [141,142], differently from its mammalian counterparts, in which one isoform is present in the cytosol and the other one in mitochondria [143]. The other enzymes involved to polyamine biosynthesis are cytosolic. Therefore, Arg seems to be taken up from the extracellular medium, converted into Orn within glycosomes, after which Orn goes back to the cytosol where it becomes a precursor in polyamine biosynthesis. However, when *Lm*ARG is expressed without its glycosomal-targeting signal, it localizes as an active enzyme to the cytosol where it is able to restore the synthesis of polyamines in ARG-knockout cell lines of *L. mexicana* promastigotes [141]. In *L. major* and *L. tropica*, expression and enzymatic activity of *Lm*ARG were found to be higher in promastigote cells during their logarithmic growth than in the stationary phase [144]. Promastigotes of *L. mexicana* and *L. donovani* ARG-null mutants are auxotrophic for polyamines and require supplementation of Orn or putrescine to grow. However, deletion of ARG had no effect on amastigotes proliferation in spleens of infected mice [141,145]. Interestingly, transcriptomics and metabolomics analyses of promastigote and amastigote ARG-null mutants revealed a tight regulation of the expression of genes associated with Pro and polyamine metabolism, as well as the levels of metabolites associated with this pathway. These results strongly suggest a metabolic regulon controlled by ARG expression [82].

*T. cruzi* and *T. brucei* use Arg in different ways than *Leishmania* spp. In the case of *T. brucei*, a metabolomics analysis of BSF showed the lack of ARG activity [88]. Additionally, the only gene encoding a putative ARG encodes an enzymatically non-functional version of ARG [146], pointing to the absence of a canonical mechanism to produce Orn from Arg. This fact would explain the relevance of Orn uptake by AAT10-1 and AAT2-4 in this parasite [46]. Interestingly, it has been shown that both parasites possess an Arg kinase (ArgK, EC 2.7.3.3) that can reversibly phosphorylate Arg to phospho-Arg (P-Arg) using ATP. P-Arg contributes to the management of the energetic metabolism in these parasites, acting like an energy backup when restoration of ATP levels is needed [51,147]. Furthermore, by measuring Arg uptake and the expression and activity levels of ArgK in different life-cycle stages, a connection between Arg metabolism and cell proliferation has been proposed for *T. cruzi* [148,149]. In *T. brucei* there are three isoforms of ArgK, being localized in the flagellum (*Tb*AK1), the mitochondrion (*Tb*AK2), and the cytosol (*Tb*AK3). Although all isoforms are expressed throughout the life cycle of these parasites, higher ArgK activities have been detected in the BSF, suggesting a major role of P-Arg in the energy metabolism of this life-cycle stage [150]. More recently, it has been shown that the flagellar isoform of ArgK is essential for *T. brucei* infectivity of the tsetse fly, although the exact mechanism still remains to be elucidated [151].

### 3.4. Branched-Chain Amino Acids

**Figure 4:**
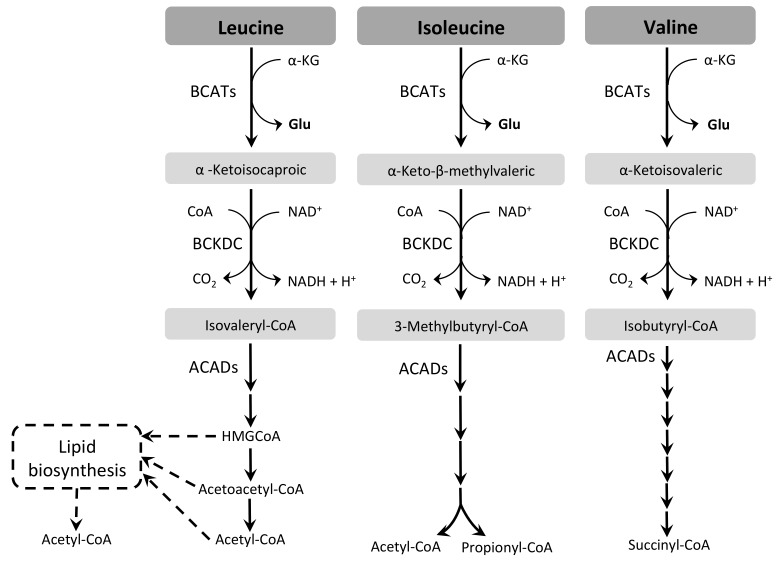
Metabolism of BCAAs. **Enzymes:** BCATs: branched-chain aminotransferases (EC 2.6.1.42); BCKDC: branched-chain α-keto acid dehydrogenase complex (E1: EC 1.2.4.4; E2: EC 2.3.1.168 and E3: EC 1.8.1.4); ACADs: acyl-CoA dehydrogenases (EC 1.3.8.7). **Metabolites:** α-KG: α-ketoglutarate, Glu: L-glutamate, CoA: coenzyme A.

Ile, Leu, and Val, the BCAA are biosynthesized only by plants and fungi among the eukaryotic organisms [83,84]. Indeed, there is evidence that BCAA are also essential in the TriTryp parasites. However, they seem to be able to catabolize them all [85,89,152,153,154]. The catabolic pathway of BCAA involves several enzymatic steps, some in common for all three amino acids and others specific for some of them. The first enzymatic reaction consists of the deamination of the BCAA producing the derived 2-oxo acid of the corresponding amino acid: α-ketoisocaproic acid, α-keto-β-methylvaleric acid, and α-ketoisovaleric acid are produced from Leu, Ile, and Val, respectively. Usually, in eukaryotes this deamination is catalyzed by specific dehydrogenases, producing NH_3_/NH_4_^+^ (ValDH, EC 1.4.1.8; LeuDH, EC 1.4.1.9) [155,156] or by branched-chain aminotransferases (BCATs, EC 2.6.1.42), pyridoxal phosphate-dependent enzymes that usually use α-KG as the acceptor for the amino group (leading to the formation of Glu) [157,158]. The BCATs might be located in the cytosol and the mitochondrion. The following enzymatic step is performed by a mitochondrial dehydrogenase complex, named branched-chain α-keto-acid dehydrogenase complex (BCKDC), which is constituted by three enzymes: E1, E2, and E3 (EC 1.2.4.4; EC 2.3.1.168; EC 1.8.1.14, respectively) [83]. The complex catalyzes the oxidative and irreversible decarboxylation of the three BCKDC, producing the acetyl-CoA derivatives isovaleryl-CoA, 3-methylbutyryl-CoA, and isobutyryl-CoA, and also NADH and CO_2_. Remarkably, all these products can be substrates of acyl-CoA dehydrogenases (ACADs, EC 1.3.8.7) as happens with the acyl-CoA derived from the β-oxidation of fatty acids in the mitochondrial matrix [159,160,161]. From this step forward, the pathways resemble some of those of fatty-acid oxidation, resulting in end products that can be introduced into the TCA cycle. The complete oxidation of Leu generates acetoacetate and acetyl-CoA (both ketogenic compounds), Val generates succinyl-CoA (glucogenic), and Ile produces propionyl-CoA and acetyl-CoA (glucogenic and ketogenic compounds, respectively) [83] (Figure 4).

The first studies on BCAA catabolism in TriTryps were performed on *T. cruzi* and *L. donovani*, demonstrating that the insect forms are able to metabolize Leu through the TCA cycle [162,163]. Leu is the most studied BCAA in TriTryps, since it has been described as a precursor of sterol and fatty-acid biosynthesis in *Leishmania* spp., *T. cruzi*, and *T. brucei*, although the latter species prefers acetate as a precursor [164,165,166,167]. Noteworthy, glucose and Thr are also main carbon sources used by *T. brucei* PCF for acetyl-CoA and lipid biosynthesis [94,168,169].

*BCAT* genes have been predicted in *T. brucei* and *Leishmania* spp. genomes [20]. Surprisingly, the *T. cruzi* genome lacks a putative *BCAT* gene, although *T. cruzi* epimastigotes are able to fully oxidize BCAA [170]. This dilemma was recently solved by showing the unusual BCAT activity displayed by the *T. cruzi* TAT and ALAT [171]. Putative genes encoding BCKDC and ACAD are also present in the TriTryp genome database, which were recently characterized in our laboratory (Manchola, N. C. et al., Ornitz, O. O. S. et al., unpublished data). 

### 3.5. Asparagine and Aspartate

**Figure 5:**
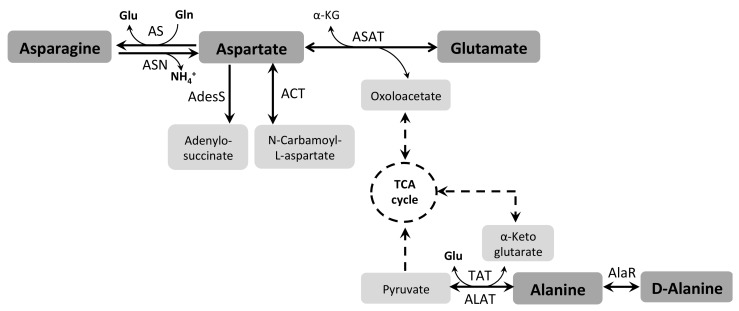
Metabolism of asparagine, aspartate and alanine. **Enzymes:** AS: asparagine synthetase (EC 6.3.1.1); ASN: asparaginase (EC 3.5.1.1); AdesS: adenylosuccinate synthetase (EC 6.3.4.4); ACT: aspartate carbamoyl transferase (EC 2.1.3.2); ASAT: aspartate aminotransferase (EC 2.6.1.1); ALAT: alanine aminotransferase (EC 2.6.1.2); TAT: tyrosine aminotransferase (EC 2.6.1.5); AlaR: alanine racemase (EC 5.1.1.1). **Metabolites:** α-KG: α-ketoglutarate, Glu: L-glutamate, Gln: L-glutamine.

As mentioned above, Asp is transported into *T. cruzi* epimastigote and trypomastigote cells [59] and is important for the metacyclogenesis process [172]. Moreover, Asp is converted to oxaloacetate by the enzyme aspartate aminotransferase [118] (ASAT, EC 2.6.1.1), and participates in ATP production through the TCA cycle and subsequent oxidative phosphorylation [93]. *T. cruzi* and *T. brucei* present two isoforms of the enzyme Asp aminotransferase, one being cytosolic (cASAT) and the other one mitochondrial (mASAT). In *T. brucei*, the cASAT is constitutively expressed and mASAT is down-regulated in BSF, whereas in *T. cuzi* the mASAT is expressed along the whole life cycle and the cASAT is specifically expressed during the mammalian stages [173,174]. Asp can also be synthesized from the TCA cycle intermediate oxaloacetate and by deamination of Asn (Figure 5). 

It has been shown that, in *T. brucei* BSF, Asp is a precursor of pyrimidine biosynthesis through the enzymes Asp carbamoyl transferase (ACT, EC 2.1.3.2) and adenylosuccinate synthetase [175] (AdesS, EC 6.3.4.4). Asp can also be converted into Asn by the enzyme Asp ammonia ligase (EC 6.3.1.1) which catalyzes the formation of Asn from Asp and NH_4_^+^, or Asn synthetase (AS, EC 6.3.1.1), that could use either NH4^+^ or Gln (EC 6.3.5.4) as nitrogen donors, both reactions being ATP-dependent. Additionally, this parasite becomes auxotrophic for Asn when AS expression is knocked down [176].

*Leishmania* is able to synthesize both Asp and Asn from oxaloacetate by a mitochondrial ASAT and an AS [102]. Asp can support growth and Asn is non-essential when the parasites are cultivated in the presence of glucose [85]. Similarly as to what occurs in *T. cruzi* and *T. brucei*, *Leishmania* also has an active AS which can use NH_4_^+^ or Gln as nitrogen donors. This enzyme is not essential for parasite growth, infectivity, or survival in normal cultivation conditions [177]. More recently, an asparaginase (ASN, EC 3.5.1.1) has been characterized in *Leishmania*. This enzyme converts Asn into Asp and ammonia [178]. ASN was also demonstrated to be involved in conferring resistance to the drug amphotericin B in both amastigote and promastigote forms grown in axenic conditions [179]. 

### 3.6. Cysteine and Methionine

**Figure 6:**
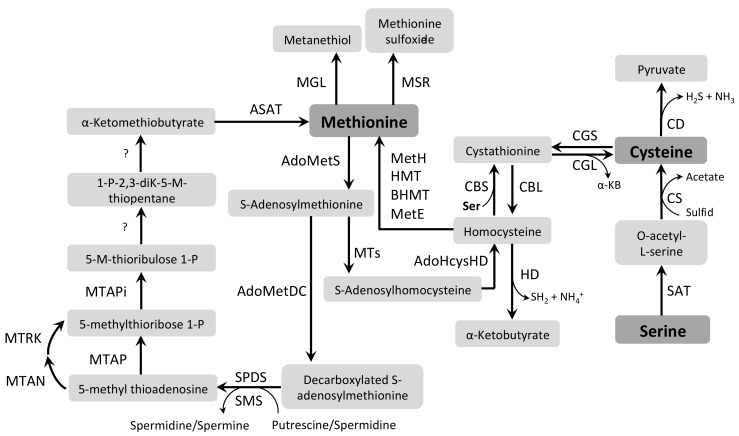
Metabolism of methionine and cysteine. **Enzymes:** AdoMetS: S-adenosylmethionine synthetase (EC 2.5.1.6); MTs: methyltransferases (EC 2.1.1.-); AdoHcysHD: S-adenosylhomocysteine hydrolase (EC 3.3.1.1); CBS: cystathionine β-synthase (EC 4.2.1.22); CBL: cystathionine β-lyase (EC 4.4.1.8); CGL: cystathionine ɣ-lyase (EC 4.4.1.1); CGS: cystathionine ɣ-synthase (EC 2.5.1.48); BHMT: betaine-homocysteine S-methyltransferase (EC 2.1.1.5); HMT: homocysteine S-methyltransferase (EC 2.1.1.10); MetH: methionine synthase (EC 2.1.1.13); MetE: methionine synthase (EC 2.1.1.14); AdoMetDC: AdoMet decarboxylase (EC 4.1.1.50); SPDS: spermidine synthase (EC 2.5.1.16); SMS spermine synthase (EC 2.5.1.22); MTAP: 5-methylthioadenosine phosphorylase (EC 2.4.2.28); MTAPi: 5-methylthioribose 1-phosphate isomerase (EC 5.3.1.23); MTAN: 5-methylthioadenosine nucleosidase (EC 3.2.2.16); MTRK: 5-methylthioribose kinase (EC 2.7.1.100); ASAT: aspartate aminotransferase (EC 2.6.1.1); MGL: methionine ɣ-lyase (EC 4.4.1.11); MSR: methionine sulfoxide reductase (EC 1.8.4.13/1.8.4.14); CD: cysteine disulfurase (EC 2.8.1.7); CS: cysteine synthase (EC 2.5.1.47); SAT: serine acetyltransferase (EC 2.3.1.30); HD: homocysteine desulfurase (EC 4.4.1.2). **Metabolites:** α-KB: α-ketobutyrate, Ser: L-serine. Question mark (?) indicates unknown step.

Met metabolism is involved in the biosynthesis of Cys, phospholipids, and polyamines [173,180]. Catabolism and biosynthesis of Met are connected by two regeneration cycles. The first catabolic step is common to both Met regeneration cycles: it produces S-adenosylmethionine (AdoMet) from Met and ATP by S-adenosylmethionine synthetase (AdoMetS, EC 2.5.1.6), also named Met adenosyltransferase. From this point, AdoMet can be a methyl donor for several methyltransferases, or it can be decarboxylated. Despite the large variety of specific methyltransferases (MTs, EC 2.1.1.-), such as Cys 5-methytransferase (CMT, EC 2.1.1.37), the major common byproduct is S-adenosylhomocysteine [180,181] (AdoHcys). From AdoHcys formation, the catabolism of Met can generate propionyl-CoA (a precursor of succinyl-CoA) via the degradation to α-ketobutyrate (α-KB) [182,183], through five enzymatic steps. The last three steps are performed by S-adenosylhomocysteine hydrolase (AdoHcysHD, EC 3.3.1.1), cystathionine β-synthase (CBS, EC 4.2.1.22), and cystathionine γ-lyase (CGL, EC 4.4.1.1), producing, respectively, homocysteine, cystathionine, and α-KB together with Cys. One of the Met regeneration cycles is completed by the conversion of homocysteine to Met by four enzymes, i.e., betaine-homocysteine S-methyltransferase (BHMT, EC 2.1.1.5), homocysteine S-methyltransferase (HMT, EC 2.1.1.10), and either Met synthase (MetH, EC 2.1.1.13) or cobalamin-independent Met synthase [180,184] (MetE, EC 2.1.1.14). Homocysteine itself can be catabolized by homocysteine desulfurase (HD, EC 4.4.1.2), forming sulfide, α-KB, and NH_4_^+^ [185] (Figure 6). 

As already mentioned, AdoMet can also undergo decarboxylation, initiating the other Met regeneration cycle [185,186]. This decarboxylation is performed by AdoMetDC producing decarboxylated AdoMet (DecAdoMet), which is an aminopropyl donor in the polyamine biosynthesis [187]. In mammals, this catalytic step is rate-limiting in the polyamine formation [188]. The aminopropyl moiety can be transferred to putrescine or spermidine yielding, respectively, spermidine or spermine by SPDS or SMS [180]. The reactions catalyzed by the latter two enzymes also produce 5-methyl thioadenosine, which is converted directly to 5-methylthioribose 1-phosphate by 5-methylthioadenosine phosphorylase (MTAP, EC 2.4.2.28), or through two steps performed by 5-methylthioadenosine nucleosidase (MTAN, EC 3.2.2.16) and 5-methylthioribose kinase (MTRK, EC 2.7.1.100). Next, 5-methylthioribulose 1-phosphate is formed from 5-methylthioribose 1-phosphate by 5-methylthioribose 1-phosphate isomerase (MTAPi, EC 5.3.1.23). Two more steps lead to the production of α-ketomethiobutyrate (KMTB); the intermediates have been identified, but the enzymes/reaction mechanisms involved have not yet been fully elucidated [185]. Finally, the regeneration of Met from KMTB occurs through the activity of an aromatic amino acid transaminase [189]. In addition, Met can be catabolized to methanethiol and α-KB by Met γ-lyase (MGL, EC 4.4.1.11) [185,186] and to Met sulfoxide (MetSO) by Met sulfoxide reductases (MSRs): Met (S)-S-oxide reductase (MSR-A, EC 1.8.4.13) and Met (R)-S-oxide reductase (MSR-B, EC 1.8.4.14), which produce two enantiomers, R-MetO and S-MetO, respectively [190] (Figure 6).

The first step of Met catabolism has been extensively studied in *L. infantum*. AdoMetS activity was measured in promastigotes and shown to be higher in lag and early-log phases of growth [191]. The genomic DNA of the parasite has two identical genes encoding AdoMetS. Through kinetic studies made with the heterologously-expressed enzyme it was shown that it was insensitive to the expected allosteric inhibition by AdoMet [192]. Two other enzymatic steps in Met metabolism were also studied in *Leishmania* spp.: (i) the decarboxylation of AdoMet, and (ii) the Met oxidation. The decarboxylation of AdoMet is performed by an AdoMetDC. In *Leishmania* spp., one gene encoding this enzyme was found, but, additionally, a copy annotated as a putative AdoMetDC-like proenzyme (AdoMetDCL) could be found, as in other trypanosomatids [193,194,195]. In *L. donovani* the knockout of the AdoMetDC gene led to spermidine auxotrophy [196]. In addition, structural and functional characterization of AdoMetDCL in *L. donovani* was performed, evidencing that it binds AdoMet and putrescine and may play an essential role in polyamine biosynthesis [195]. The oxidation of Met is performed by a MRS-A. This enzyme has been characterized in *L. major* where the genome encodes one functional copy for the cytosolically located *Lm*MRS-A. Parasites knocked out for *Lm*MRS-A exhibited increased sensitivity to H_2_O_2_. However, this protein is not essential for pathogenesis *in vivo* [197].

Initially, in BSF of *T. b. brucei*, different Met catabolic enzymes were identified: activities of AdoMetDC [198], AdoMetS, AdoHcysHD, CBS, and several MTs (guanidinoacetate N-methyltransferase-GAMT, EC 2.1.1.2; Gly N-methyltransferase-GNMT, EC 2.1.1.20; HMT; and MetH) were described. Yarlett and Bacchi also demonstrated that the AdoMet concentration is 50-fold higher in BSF isolated from rats treated with DFMO than from untreated rats [199]. Subsequently, the kinetic characterization of AdoMetS activity was performed in these parasites, evidencing the presence of two isoforms and a weak inhibition by the AdoMet product when compared to the corresponding mammalian enzyme [200]. Another study by Bacchi and coworkers [201] showed that these parasites are able to metabolize Met into intermediates of the polyamine and methyltransferases pathways. The AdoMetDC activity was initially described as being insensitive to putrescine and Mg^2+^ [198]. However, Tekwani et al. [202] showed a strong activation by putrescine when cell-free extracts were dialyzed, similarly as to what occurs with AdoMetDC from mammalian cells. Like other trypanosomatids, *T. brucei* has a gene encoding an active AdoMetDC and a catalytically-inactive version of AdoMetCD (“prozyme”) which is able to form a high-affinity heterodimer stimulating the AdoMetDC activity [193,203]. In *T. brucei* the next (and last) metabolic step after AdoMet decarboxylation can be performed by the ASAT, which also has catalytic activity of an ALAT [173,174,189]. 

Met oxidation was also investigated in *T. brucei*: cloning, bacterial expression, and characterization of a MSR-A has been reported that is expressed in both BSF and PCF stages [204]. Recently, two more MSRs from *T. brucei* were characterized, a cytosolic one homologous to the MSR-A family, and a mitochondrial one, with sequence motifs typical of MSR-Bs. Interestingly, a reduced expression of *Tb*MSR-A gave rise to an increased susceptibility to exogenous H_2_O_2_, while elevated expression levels resulted in more resistance to oxidative imbalance [205].

In *T. cruzi*, the first study of Met metabolism reported the cloning of putative genes of AdoMetDC and AdoHcysHD, as well as the expression and kinetic characterization of their proteic products [206,207]. Like in *T. brucei*, TcAdoMetDC is stimulated by putrescine [206] and by a prozyme [203]. Still in the same way as in *T. brucei*, the cytosolic TcASAT regenerates Met from KMTB [174]. Lastly, two genes encoding TcMSR-As were cloned and the proteins expressed and characterized. The MSR-A activity was also detected in cytosolic extracts of replicative stages (epimastigotes and amastigotes). Furthermore, the overexpression of one of the isoforms in epimastigotes increased the resistance of these cells to oxidative imbalance generated by exogenous H_2_O_2_ [204]. 

Beyond its role as a sulfur group-containing building block for proteins, Cys is crucial for the biosynthesis of the reducing agent trypanothione in trypanosomatids. In fact, Cys has been shown to be essential for cell viability in BSF *T. brucei* and addition of this amino acid to the culture medium enabled, axenically, culture of this life-cycle stage [36,208,209]. In contrast, *L. major* and *L. mexicana* promastigotes are able to grow in the absence of Cys [85,210], suggesting that a Cys biosynthesis pathway probably exists in these organisms.

As mentioned above, Cys can be synthesized by using Met as a precursor via the reverse transsulfuration pathway. This pathway involves the enzymes CBS and CGL. Alternatively, Cys biosynthesis can also be achieved via Ser acetyltransferase (SAT, EC 2.3.1.30) which catalyzes the conversion of Ser to O-acetylserine (OAS) and Cys synthase [211] (CS, EC 2.5.1.47) (Figure 6), which catalyzes the addition of a sulfide group to O-acetylserine (OAS). *T. cruzi* and *L. major* seem to be the only unicellular organisms studied to date that contain both active pathways and the biological relevance of this redundancy for Cys biosynthesis remains to be elucidated. The characterization of these pathways in *T. cruzi* has shown that CBS has a higher activity in epimastigotes, whereas CS is active both in epimastigotes and amastigotes. Regarding their expression, both CBS and CGL have been shown to be more expressed in the insect-stage epimastigote whilst CS is more abundant in the mammalian-stage amastigote, suggesting a developmental regulation of the reverse transsulfuration pathway in this organism [212,213]. Noticeably, the genome of *T. brucei* contains putative coding sequences only for CBS and CGL, but the reverse transsulfuration pathway remains uncharacterized in this organism.

In *L. braziliensis* amastigotes, CS levels and activity were increased together with CBS and CGL, whereas CBS is more active in promastigotes. The overexpression of either CBS or CS in *L. braziliensis* promastigotes increased the resistance of these parasites to oxidative imbalance, in agreement with the role of Cys as an anti-oxidant and its participation as a precursor of trypanothione biosynthesis [214]. CGL was also characterized in *L. major* and *L. mexicana*, where it was shown to have a cytosolic localization in promastigotes [215]. SAT and CS are also present in the cytosol, forming a complex that would have an increased activity compared to the individual enzymes, as suggested by *in vitro* studies in which *L. major* and *L. donovani* CS stimulate their respective SAT activities [179,210,216], similarly as previously observed in plants [217]. 

Cys can be also catabolized through the action of Cys desulfhydrase (CD, EC 2.8.1.7), which converts Cys into H_2_S, NH_3_, and pyruvate. This enzyme has only been characterized in *L. major*, being able to specifically use Cys as a substrate [216]. Cys can also be converted back to Met via cystathionine β-lyase (CBL, EC 4.4.1.8) and cystathionine γ-synthase (CGS, EC 2.5.1.48) but, to date, this pathway has not been characterized in any of the trypanosomatids.

### 3.7. Other Amino Acids: Phenylalanine, Tryptophan, Tyrosine, Serine, Glycine, Threonine, Alanine, and Lysine

#### 3.7.1. Aromatic Amino Acids (Phe, Trp and Tyr)

**Figure 7:**
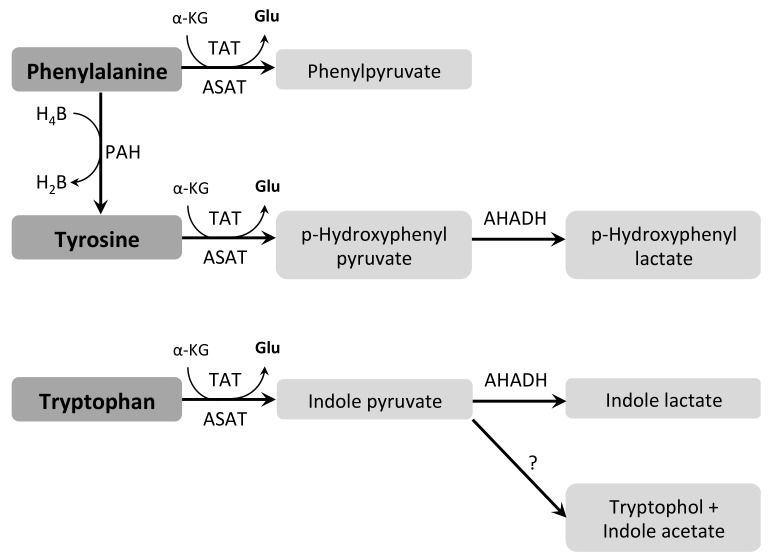
Metabolism of tyrosine, tryptophan and phenylalanine. **Enzymes:** PAH: phenylalanine hydroxylase (EC 1.14.16.1); TAT: tyrosine aminotransferase (EC 2.6.1.5); ASAT: aspartate aminotransferase (EC 2.6.1.1); AHADH: aromatic L-2-hydroxyacid dehydrogenase (EC 1.1.1.337). **Metabolites:** α-KG: α-ketoglutarate, Glu: L-glutamate, H_4_B: Tetrahydrobiopterin, H_2_B: Dihydrobiopterin. Question mark (?) indicates unknown step (adapted from [224]).

Trypanosomatids are auxotrophic for aromatic amino acids and depend on their uptake from the extracellular environment [85,91]. Once imported, these amino acids are metabolized in aromatic acids by pathways that vary among these organisms. *T. cruzi* has two enzymes involved in the catabolism of aromatic amino acids: TcTAT that produces α-oxoacids, and an aromatic L-2 hydroxyacid dehydrogenase (TcAHADH, EC 1.1.1.337) that converts α-oxoacids into α-hydroxyacids using NADH [218,219] (Figure 7). TcTAT is only expressed in epimastigotes, where it can transaminate Phe, Trp, and Tyr, as well as Ala, Leu, Ile, Val, Glu, and Met using pyridoxal phosphate (PLP) as a cofactor, with a preference for Tyr [171,218,220,221,222]. Not much is known about TcAHADH, which belongs to the malate dehydrogenases family, although it does not have any MDH activity [223]. 

In *L. donovani*, transaminases are active, having most amino acids as amino group donors and both α-KG and pyruvate as preferred acceptor co-substrates [225,226]. In addition, a broad-specificity aminotransferase (BSAT, EC 2.6.1.1) has been identified, able to transaminate all aromatic amino acids, as well as Asp, Met, Leu and, with a lower activity, Ala in *L. mexicana* [227,228]. Similarly to *T. cruzi*, *Leishmania* spp. also have a TAT, which is able to transaminate aromatic amino acids and Met using pyruvate as an amino group acceptor. However, it does not have ALAT activity [129]. There is no AHADH in *Leishmania* spp. in agreement with the absence of aromatic products excreted in culture media [224,228]. Finally, *Leishmania* contains a Phe hydroxylase (PAH, EC 1.14.16.1) that can convert Phe into Tyr using tetrahydrofolate as a co-substrate [229].

In *T. brucei*, conversion of Phe into phenylpyruvate, as well as Tyr into p-hydroxyphenylpyruvate and p-hydroxyphenyllactate, the latter reaction being NADH-dependent, has been demonstrated. Indoleacetate, indolepyruvate, and tryptophol have been detected as products of Trp catabolism [230,231,232]. Additionally, several studies reported that products of aromatic amino-acid metabolism were increased in urine and blood of animals infected with *T. b. gambiense* and *T. b. evansi* [233,234,235]. Furthermore, it has been demonstrated that indolepyruvate can modulate the immune response in infected mice [236], which points to a role for aromatic amino acids in the successful establishment of an infection by *T. brucei*.

So far, no homolog of TAT has been found in *T. brucei*. Instead, it has been recently shown that the aromatic ketoacids resulting from Phe, Tyr, and Trp metabolism are products of the activity of the cytosolic ASAT in BSF *T. brucei* [174]. Furthermore, aromatic amino acids play an important role in Met recycling, where they act as the major amino group donor to α-ketomethiobutyrate to yield Met [173,189]. 

#### 3.7.2. Glycine and Serine

**Figure 8:**
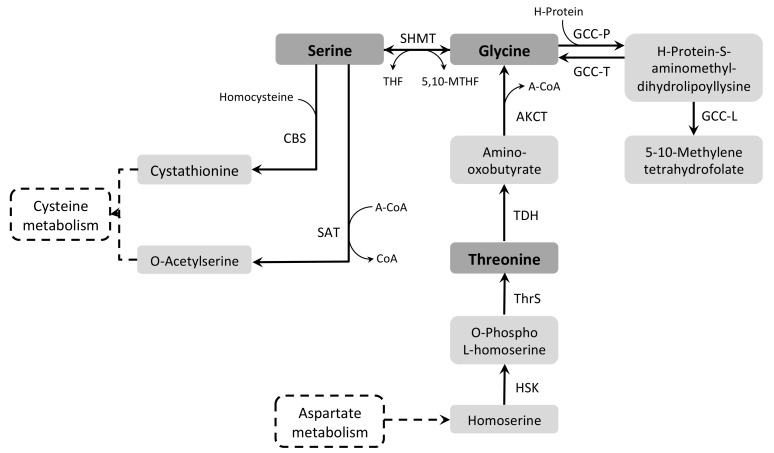
Metabolism of serine, glycine and threonine. **Enzymes:** CBS: cystathionine β-synthase (EC 4.2.1.22); SAT: serine acetyltransferase (EC 2.3.1.30); SHMT: serine hydroxymethyltransferase (EC 2.1.2.1]; TDH: threonine dehydrogenase (EC 1.1.1.406]; ThrS: threonine synthase (EC 4.2.3.1); HSK: homoserine kinase (EC 2.7.1.39); AKCT: 2-amino-3-ketobutyrate CoA-transferase (EC 2.3.1.29); GCC-P: glycine decarboxylase (EC 1.4.4.2); GCC-T: tetrahydrofolate aminomethyltransferase (EC 2.1.2.10); GCC-L: dihydrolipoamide dehydrogenase (EC 1.8.1.4). **Metabolites:** A-CoA: acetyl coenzyme A, CoA: coenzyme A, THF: tetrahydrofolate, 5,10-MTHF: 5-10-methylene tetrahydrofolate.

Both Ser and Gly metabolic pathways can contribute to folate metabolism, as well as to Cys and Met synthesis. In the former process, tetrahydrofolate (THF) is used as a co-substrate in a reversible reaction with Ser catalyzed by Ser hydroxymethyltransferase (SHMT, EC 2.1.2.1) resulting in 5,10-methylene tetrahydrofolate (5,10-MTHF) and Gly [237]. 5,10-MTHF is an important precursor in the thymidylate and Met synthesis, and it can also be synthesized in the mitochondrion from Gly and THF through the action of the Gly cleavage complex (GCC) [238] (Figure 8).

The first evidence that Ser could be metabolized in trypanosomatids was provided by Hampton, when analyzing the incorporation of radioactivity from both uniformly-labeled Ser and Ser-1-^14^C. Labeled carbons were incorporated in all fractions except nucleic acids. In addition, a large production of radio-labeled CO_2_ was detected when *T. cruzi* epimastigotes were incubated with Ser-1-^14^C [47]*.* Cys, Ala, Gly, as well as Glu and Asp, can also be products of Ser metabolism [239]. Later, SHMT activity was demonstrated in *T. cruzi, Trypanosoma rangeli* and *Leishmania* spp., with *T. cruzi* having the highest activity of this enzyme [240].

Two isoforms of SHMT have been characterized in *Leishmania*, one being cytosolic and the other one mitochondrial [241]. In the presence of Ser, both isoforms have been shown to be dispensable for cell growth in *L. major*. However, gene knock-out of one or both of the SHMT isoforms is lethal in the absence of Ser [242].

The *T. cruzi* genome contains a single *SHMT* gene, which encodes the cytosolic isoform and uses pyridoxal phosphate (PLP) as a cofactor [243]. In contrast, no *SHMT* gene is present in the *T. brucei* genome, which suggests that *T. brucei* depends only on GCC to synthesize 5,10-MTHF.

GCC is a complex formed by four subunits: a Gly decarboxylase that is known as P-protein (GCC-P, EC 1.4.4.2), a tetrahydrofolate aminomethyltransferase known as Gly synthase or T-protein (GCC-T, EC 2.1.2.10), a dihydrolipoamide dehydrogenase known as L-protein (GCC-L, EC 1.8.1.4), and an H-protein which contains a lipoamide prosthetic group. There are putative homologs for the four subunits of the complex encoded in the *L. major, T. brucei*, and *T. cruzi* genomes. In *Leishmania* spp., subunit P is localized in the mitochondrion and its knockout in amastigotes caused a delay in the progress of an infection in mice [244]. In *T. brucei* and *T. cruzi*, L-protein is the best-characterized GCC subunit and it catalyzes the formation of 5,10-MTHF in a NAD-dependent reaction. In *T. brucei* this enzyme is localized in the mitochondrion and, accordingly, it is more expressed in the procyclic form. However, the L-protein has been shown to be essential in both insect and mammalian stages of the parasite [245,246,247,248,249]. In *T. cruzi* this enzyme has been investigated as a potential drug target since it is inactivated by free radicals derived from phenol and its mRNA is up-regulated in cell lines resistant to benznidazole [250,251,252,253]. 

#### 3.7.3. Threonine

In *T. brucei* PCF, Thr is the most-consumed amino acid regardless of the growth conditions, with a consumption rate comparable to that of glucose [38,254]. However, Thr cannot sustain growth of the parasite *in vitro* since it is converted into other metabolites, including the excreted acetate and Gly end products, which are not or poorly used for energy production in trypanosomatids [38]. Thr is first reduced by Thr dehydrogenase (TDH, EC 1.1.1.103) into amino-oxobutyrate, which is converted into acetyl-CoA and Gly by 2-amino-3-ketobutyrate CoA-transferase (AKCT, EC 2.3.1.29). Then, acetyl-CoA is converted into acetate by acetate:succinate CoA-transferase (ASCT, EC 2.8.3.8), or acetyl-CoA thioesterase [168,255] (ACH, EC 3.1.2.3). In *in vitro* conditions, Thr, via acetyl-CoA, is the preferred source of carbon for fatty-acid and sterol synthesis [169,256]. Interestingly, expression of TDH is down-regulated in a phosphoenolpyruvate carboxykinase (PEPCK) null background probably as a consequence of the redirection of the metabolic flux towards acetate production, suggesting that a metabolite of this latter pathway participates in the control [169] (Figure 8). 

*Leishmania* spp. and *T. cruzi* genomes do not contain genes encoding enzymes for the Thr degradation pathway, suggesting that these parasites are not able to catabolize this amino acid [169]. This is consistent with Thr being one of the major components of the amino-acid pool in *L. tropica* promastigotes [257]. 

Thr can be synthesized *de novo* from Asp in a multistep pathway that involves aspartokinase (AspK, EC 2.7.2.4), Asp semialdehyde dehydrogenase (AspSD, EC 1.2.1.11), and homoserine dehydrogenase (HSD, EC 1.1.1.3), which culminates in homoserine production. Homoserine is then phosphorylated by homoserine kinase (HSK, EC 2.7.1.39) and the resulting O-phospho-homoserine is finally converted into Thr by Thr synthase (ThrS, EC 4.2.3.1) (Figure 8). *Leishmania* spp. genomes encode all enzymes of this pathway, and the conversion of Asp into Thr has been demonstrated in *L. mexicana* [258]. However, it has recently been reported that the absence of Thr in the culture medium increases the doubling time of promastigotes, whilst it decreases the cellular protein content and size, suggesting that the Thr *de novo* biosynthetic pathway is not sufficient for optimal parasite growth [85]. Interestingly, the *T. brucei* genome only encodes the last two steps of the pathway and an HSK has been characterized. Growth of the PCF HSK-null mutant is compromised in the absence of Thr and rescued by the addition of homoserine [259]. Incidentally, the tsetse fly microbiota includes two species of bacteria—*Soldalis glossinidius* and *Wigglesworthia glossinidia*—producing homoserine, which could be a source of Thr in the *T. brucei* PCF *in vivo*, explaining why the last two steps of the pathway have been retained.

#### 3.7.4. Alanine

Ala constitutes the majority of the free amino acid pool in trypanosomes [260,261] and *Leishmania* spp. [257]. It has been demonstrated in *Leishmania* that approximately 90% of the free Ala pool corresponds to the L-isomer. The remaining part is formed by D-Ala that is synthesized by the activity of the Ala racemase [262] (AlaR, EC 5.1.1.1). Putative coding sequences for Ala racemase are present in both the *T. cruzi* and *T. brucei* genomes, but they still remain uncharacterized. Extracellular Ala uptake has been demonstrated in *T. b. gambiense* [33] and *L. donovani* [78]. It can also be synthesized by different pathways in trypanosomatids. In fact, Frydman and collaborators used ^13^C-nuclear magnetic resonance to investigate Ala pools in *T. cruzi* epimastigotes. They were able to identify two distinct pools: one originated from glucose metabolism and the other one from a different source [263]. 

In addition to uptake and synthesis, Ala is also excreted by trypanosomatids [264]. Actually, the levels of this amino acid are increased in the serum, but not in tissues of mammals infected with *T. b. gambiense* and *T. brucei* [265,266,267]. In PCF *T. brucei*, Ala is the main excreted product from Pro metabolism when cells are grown in the absence of glucose. As mentioned earlier, the excreted Ala is the product of the amination of pyruvate through the action of transaminases. The main known sources of pyruvate are the conversion of malic acid through the mitochondrial or cytosolic malic enzymes or the production from phosphoenolpyruvate by pyruvate kinase [94] (PK, EC 2.7.1.40). *T. brucei* has a coding sequence for a putative mitochondrial ALAT, of which expression has been detected in both the cytosol of both the PCF and BSF. It specifically transaminates Ala using α-KG as an amino acceptor, generating pyruvate and Glu (Figure 5). Down-regulation of *Tb*ALAT induces a strong growth defect of the PCF trypanosomes in glucose-depleted conditions, which confirms that ALAT activity is essential for the viability of the parasite in the insect vector, where the availability of glucose is limited [129,268,269]. 

In *T. cruzi* and *L. donovani*, ALAT is expressed throughout all stages of the life cycle, but higher expression levels have been observed in amastigote stages [270]. TcALAT is localized in both the mitochondrion and the cytosol [129]. The poorly specific TcTAT is also localized in the cytosol and, therefore, the cytosolic pool of Ala/pyruvate might be regulated by the combined activity of these two enzymes. Furthermore, as previously mentioned, in *T. cruzi* epimastigotes Ala can also be generated as a product of Ser metabolism [239].

#### 3.7.5. Lysine

Trypanosomatids are unable to synthesize Lys and rely on uptake to obtain this amino acid [52,90,91]. Accordingly, Lys has been shown to be essential for cell growth in *Leishmania* [85].

In summary, a list of enzymes that use amino acids as substrates and their respective genes’ accession numbers in TriTryps is detailed in Table 2.

## 4. Non-Obvious Roles of Amino Acids

### 4.1. Host-Parasite Interaction

*T. cruzi*, along its life cycle, needs to adapt to different nutrients available in the different environments: the bloodstream, the intracellular environments in the different tissues it can invade in the mammalian host, and the different parts of the midgut in the insect vector. Epimastigotes in the early exponential growth phase (*in vitro*) are equipped with the complete set of glycolytic enzymes and, in fact, they obtain energy from glucose catabolism. However, it is not known if this is possible inside the insect midgut, since relevant quantities of free sugars in this environment have not been demonstrated so far [271]. However, the availability of amino acids has been well demonstrated [272,273] and the ability of epimastigotes to catabolize these metabolites is well understood, as previously described. In addition to their role in the parasite’s bioenergetics, it was demonstrated that some of these amino acids have relevance in the survival of the epimastigotes during the nutritional, redox, osmotic, and thermal stresses that they suffer in the midgut [274,275]. For example, Pro participates in the parasite’s resistance to thermal stress, while both Pro and Glu contribute to resistance to the nutritional stress and redox imbalance [276,277]. Additionally, His was shown to extend parasite survival in nutritional stress conditions, such as that experienced by epimastigotes during the colonization of the triatomine midgut [61].

Nutritional stress also plays a functional role in *T. cruzi*’s life cycle. Metacyclogenesis, i.e., the differentiation of the proliferative non-infective epimastigotes into the non-proliferative infective metacyclic trypomastigotes, is triggered when the epimastigotes are starved [172], followed by exposure to a supply of nutrients, notably some amino acids. To analyze this process in more detail, Contreras and collaborators developed a minimum medium for *in vitro* induction of metacyclogenesis. They investigated the effect of individually adding 10 different L-amino acids to artificial triatomine urine. In this study, Pro was the only amino acid able to support an approximately 90% efficient differentiation [172], although subsequent experiments showed that Gln and Asn were also able to induce metacyclogenesis, with efficiencies similar to that of Pro [278]. Interestingly, it was shown that the consumption of amino acids continued after completion of metacyclogenesis [279].

Since the next differentiation step, of the non-dividing metacyclic trypomastigotes into proliferating amastigotes occurs exclusively in the intracellular environment, invasion of host cells by the trypomastigotes should be considered a crucial step for establishing the infection in the mammal. The parasite requires energy for this host-cell invasion [280]. It was shown that this energy can be provided by Pro, its metabolite P5C and, to a lesser extent, Glu, because these compounds were shown to make the mammalian host-cells infection much more efficient [96,279].

Trypomastigote forms derived from a previous mammalian host-cell infection are, most of the time, present in the glucose-rich bloodstream, and are able to consume this compound for their energy metabolism [281]. Once the parasites establish the infection in the cytosol of the host cells as amastigotes, they go through a metabolic switch to preferably consume the intracellular pools of amino acids, mainly Pro, as carbon and energy source. In fact, the intracellular environment is poor in glucose and amastigotes are unable to take this sugar up from an environment at concentrations below the millimolar range [281], despite the fact that amastigotes can take up and metabolize glucose when available at high concentrations [282]. It was also demonstrated that amastigotes can acquire fatty acids from the host to support infection [283,284]. 

As mentioned earlier, the metabolism of amino acids occurs with the production of NH_4_^+^ as a result of the transfer of the amino group to H_2_O [185]. As *T. cruzi* does not have a functional urea cycle [113], the detoxification of the accumulated NH_4_^+^ occurs through its metabolism to form non-toxic metabolites, notably through its incorporation into α-KG to form Glu. However, a high consumption of α-KG to deal with NH_4_^+^ accumulation could compromise the functioning of the TCA cycle. Thus, α-KG is regenerated by a transamination reaction in which pyruvate is the main amino group acceptor, yielding also Ala [122]. As the accumulation of Ala can be detrimental as well, we proposed and recently demonstrated that the enzyme GS can contribute to the detoxification of NH_4_^+^. In fact, GS has its highest activity in the amastigote stage and we showed its participation in the regulation of the NH_4_^+^ release during the transient residence of *T. cruzi* in the parasitophorous vacuole [125]. The acidic pH of this vacuole is critical for the parasite differentiation from trypomastigotes to amastigotes and for the release of the amastigotes into the host-cell cytosol, a *sine qua non* condition for the establishment of the intracellular infection [285]. Moreover, it was recently shown that intracellular parasite forms possess transmembrane transporters their in acidic organelles to sequester the ammonium resulting from amino-acid metabolism. This system enhances the parasite’s resistance to starvation and osmotic stress [286]. 

The intracellular epimastigote is a transient stage between amastigote and trypomastigote, and expresses some of the trypomastigote surface proteins, probably as an early adaptation to the needs that trypomastigotes, the next stage, will face [5]. As such, its ability to transport glucose from the medium (which is, in this case, the host-cell cytosol) is not surprising [281]. However, it was shown that among all the stages analyzed so far, intracellular epimastigotes are those with the highest specific activities of Pro uptake, PRODH and P5CDH, i.e., the enzymes involved in the oxidation of Pro to Glu. This increase of Pro uptake serves probably to compensate for a decrease of the intracellular stocks of free Pro due to its catabolism [95,96,101,281]. Even more, when the infection happens in a Pro-poor environment, the total number of released trypomastigotes, as well as the differentiation from intracellular epimastigotes to trypomastigotes, are compromised, indicating that Pro is involved not only in the intracellular proliferation of the parasites, but also in their differentiation [101]. In addition, different studies suggested that the Pro-dependent enzyme TcPRAC is involved in cellular differentiation and invasion [117,287] and in the development of the parasite’s resistance to the host proteolytic enzymes through the incorporation of D-Pro into parasite proteins [288]. Remarkably, the secreted isoform of TcPRAC, an enzyme having Pro as a substrate, is an effective mitogen for host B lymphocytes, favoring parasite evasion of specific immune responses [115,116]. This activity is dependent on the exposure of a transient epitope in the ligand-free enzyme [289]. Consequently, the mitogen activity would be regulated by the availability of Pro in the mammalian host blood. 

*T. brucei* also alternates between its mammalian host and insect vector and adapts its metabolism to the availability of nutrients in these different environments. The BSF use glucose as an energy source that is abundant in the mammalian blood [30,290,291]. Interestingly, it was recently shown that, in spite of the existence of a fully-functional Gln biosynthesis pathway in long-slender BSF, the supply of external Gln is essential for the BSF’s survival and replication (Damasceno et al., unpublished data). Little is known about the differentiation process from metacyclic forms to the replicative long-slender BSF. However, some more information is available on the differentiation process from long-slender BSF to the non-proliferative and insect-infective stumpy BSF. This process is dependent on the parasitemia level and on the concentration of the stumpy-induction factor (SIF) [292]. Although still of unknown identity, it has been shown that SIF is a low molecular weight soluble molecule produced by the parasite [8]. Transformation to the stumpy form induces a partial metabolic reprogramming including the upregulation of various mitochondrial processes, like the induction of ASCT expression with consequent acetate production. This likely creates a pre-adaptive condition to the next stage: the infection of the insect vector [293]. Within approximately 15 minutes after the tsetse has taken up stumpy BSF parasites during the blood meal, glucose from the host’s blood has been consumed [294]. In the midgut of the fly, Pro, the most abundant amino acid in the tsetse flies which energetically supports the flight process [268], becomes the main energy source for PCF, as well as, to a lesser extent, Thr. In fact, the need of a fully-functional Pro metabolic pathway for *T. brucei* survival in the insect’s midgut and to support establishment of the fly’s infection was recently shown [38,39,94]. Similarly, *in vitro* transformation from BSF to PCF involves addition of citrate/cis-aconitate to the differentiation medium, which lacks glucose and is rich in Pro and Gln, and a change in temperature, which, together, stimulate mitochondrial development, including the expression of TCA cycle enzymes [295,296].

*Leishmania* spp. classically present two stages: amastigote (in the mammalian host) and promastigote (in the insect vector). Proliferative promastigotes, in turn, can differentiate into non-proliferative, infective metacyclic promastigotes, which are morphologically similar to the proliferative ones [297]. The exponentially-growing promastigote expresses an enzymatic arsenal to metabolize disaccharides, probably an adaptation to the diet of sandflies, which can feed on nectar and aphid honeydew. Promastigotes face varying environmental conditions, like the physical and chemical changes due to, respectively, the temperature and the nutritional habit of the sandfly [298]. Comparative proteomic and metabolomic analyses of a mutant promastigote line with inability to transport glucose suggest that Pro is a main carbon source used for energy metabolism and gluconeogenesis [103]. The amastigote is the proliferative form and replicates inside the phagolysosomal compartment of macrophages using mainly sugars as energy source, although it presents a reduced uptake and rate of metabolism when compared to the promastigote form [299].

In fact, *Leishmania* spp. differentiate from the extracellular procyclic promastigotes to metacyclic promastigotes in the sandfly and then to the intracellular amastigotes in the mammalian host. Similar to *T. cruzi*, this transformation means an extreme change in environment, from the sand fly midgut to the acidic interior of the phagolysosome. This process is accompanied by a metabolic switch in which the parasite changes from the high use of sugars and amino acids as carbon and energy sources to a form with a globally-reduced metabolism still based on the preferential use of sugars [300]. In fact, the use of amino acids available in the phagolysosome appears to be detrimental, since it would necessitate increased activity of the TCA cycle and respiratory chain resulting in oxygen stress for which the parasites are highly susceptible in this environment [301].

*In vitro* differentiation of *Leishmani*a spp. involves changes in temperature and pH [302], but the function of amino acids in inducing cell differentiation is still to be elucidated. Diaz and collaborators investigated the role of the L-amino acids Ala, Cys, Met, Glu, Gln, Pro, and Trp in inducing chemotaxis, which has been proposed to be essential for cell differentiation. In this study, Glu, Gln, Met, and Trp had both positive and negative effects on chemotaxis depending on the amino acid concentration, suggesting the existence of some sort of sensing mechanism in *Leishmania* [303].

Arg plays a crucial role in the host-parasite interplay [304,305]: a mechanism in which *Leishmania* senses extracellular levels of Arg has been shown to impact on parasite viability inside the macrophage. The role of Arg during mammalian host-cell infection was studied in some detail. Arg is the substrate of the inducible nitric oxide synthase (iNOS), a major effector of the immune system producing the potent microbicidal NO, and which has an increased expression in activated macrophages [306]. Arg is also a substrate for the enzyme ARG which, in both amastigotes and promastigotes, is found within glycosomes, suggesting that the pool and trafficking of Arg is under control in these parasites [307]. Infection with *L. major* resulted in an additional increase of the parasite’s Arg catabolism through ARG, producing two effects: diminishing the availability of free Arg as a substrate of the iNOS, and re-directing this metabolite to the production of Orn and polyamines in the host cells. Interestingly, upon diminished levels of Arg, the parasite upregulates the expression of the Arg “transceptor” AAP3, to increase the uptake of the amino acid and compensate for the effect of its diminished levels inside the host-cells [80,308,309]. In summary, inhibition of arginase activity results in diminished Arg catabolism, decreased polyamine synthesis, and subsequently-reduced parasite growth [310], whereas at the host side the amino acid Arg is the substrate to produce nitric oxide for combating the parasite [305,309,311,312]. This metabolic balance can decide between death or survival of the parasite.

### 4.2. Role of Amino Acids in the Regulation of Autophagy and Apoptosis

As part of the attention being paid to “non-canonical roles” (i.e., beyond their involvement in the cell bioenergetics or protein synthesis) of amino acids in trypanosomatids, efforts have been made to elucidate their possible participation in regulating processes in these organisms, such as autophagy and cell death. Schmidt and Butikofer used PCF *T. brucei* expressing GFP-tagged *Tb*Atg8.1 and *Tb*Atg8.2 to investigate the conditions in which autophagy is induced in this organism. Atg8 is a cytosolic protein that, upon initiation of the autophagy process, is cleaved and modified by the addition of a phosphatidylethanolamine (PE) anchor. This modification relocates Atg8 to the developing phagophore membrane that will give rise to the autophagosomes, which will eventually fuse with the lysosome [313,314,315]. The authors found that when PCF experiences nutrient starvation, *Tb*Atg8.1-GFP and *Tb*Atg8.2-GFP presented a punctal appearance, spread throughout the cytoplasm. However, addition of His prevented the relocation of the fluorescently-tagged proteins to puncta and autophagy [316]. As previously mentioned, *T. brucei* does not have the canonic His degradation pathway and, although the mechanism by which this amino acid protects trypanosomes from autophagy is still to be elucidated, it is very likely that it is a direct effect of His and not of a product of its catabolism. Conversely, among the amino acids are not only those that protect against autophagy, but also ones that, by the trypanosome’s starvation for them, induce the process, similarly to what has been reported for other eukaryotes. For example, He and collaborators described that amino acid starvation can trigger autophagy independently of the AMP-activated-Protein-Kinase-mediated energy sensing mechanisms [317,318]. Further studies are required to unravel the details of the pathways involved. 

A more explicit mechanism has been proposed to explain the role of Arg in protecting *T. cruzi* from apoptosis. Piacenza and collaborators found that Arg prevents the formation of patterns associated with the programmed cell death process as DNA fragmentation and inhibition of [^3^H]-thymidine incorporation when epimastigotes are exposed to apoptotic stimuli. Furthermore, Arg stimulates the mammalian host’s production of NO, which is a known apoptosis regulator [319] and polyamine synthesis that sustains parasite proliferation [320]. Similarly, the absence of Arg in culture media has been associated with oxidative stress, increase in reactive oxygen species production, decrease in polyamine synthesis, and induction of programmed cell death in *L. donovani* promastigotes [321].

### 4.3. Osmotic Control by Amino Acids

Amino acids, besides being precursors of essential compounds, such as polypeptides and polyamines, also function as osmolytes used by the TriTryps to cope with the osmotic stress that they face along their life cycles. When insect forms colonize the digestive tract of their invertebrate hosts, they encounter extreme fluctuations in osmolarity caused by the regular feeding of the bugs and, particularly in the case of metacyclic forms of *T. cruzi*, by the composition of the triatomine rectal contents [322,323,324,325]. In the vertebrate hosts, the parasites still face osmotic fluctuations, since different tissues are exposed to high or low osmolarity fluids in their physiological or pathological conditions [326,327]. Therefore, osmoregulation is essential for the completion of the biological cycle by the TriTryps.

Osmotic control in trypanosomatids is mainly performed by two types of organelles: the acidocalcisomes in all trypanosomatids and in *T. cruzi* the contractile vacuole complex [328,329]. Acidocalcisomes are electron-dense acidic organelles that are rich in calcium and polyphosphates [330]. The contractile vacuole complex is a bipartite structure composed of a network of microtubules and vesicles named the spongiome, surrounding a central vacuole, or bladder [331]. This bladder swells when it accumulates excess water from the cytosol, which is eventually expelled from the cell after the bladder comes into contact with the plasma membrane [332]. In *T. cruzi*, the hydrolysis or synthesis of polyphosphates inside the acidocalcisomes occurs in response to hypoosmotic or hyperosmotic stress conditions, respectively [333]. In *L. major* the acidocalcisomal content also changes under hypoosmotic stress, changing its levels of sodium and chlorine [334]. In *T. brucei*, the role of acidocalcisomes in osmoregulation has been demonstrated in a mutant cell line deficient in polyphosphate production that became sensitive to osmotic stress [335]. 

Pools of amino acids in TriTryps also provide a reservoir of osmolytes for cells to use during osmotic stress. Under hypoosmotic stress, amino acids are mobilized for an intensive efflux to exhibit a regulatory volume response [336,337,338]. This efflux occurs through an anion channel, mobilizing uncharged and acidic amino acids, a mechanism that accounts for approximately 50% of the regulatory volume decrease [328]. Epimastigote, amastigote, and trypomastigote forms of *T. cruzi* released Glu, Gly, and, mainly, Ala and Pro [337], while promastigotes of *L. major* released Glu, Gly, Ser, and mainly Ala, which is also the major amino acid released by amastigotes of *L. donovani* [336,338]. The importance of amino acids as osmolytes was also evidenced in hypoosmotic and hyperosmotic stresses, by noticing an increase of the catabolism or biosynthesis under the respective stress conditions that serves to maintain the cytosolic ionic strength [339,340].

More recently, Inbar et al. have shown that Pro and Ala act differently in the face of hypoosmotic stress in *L. donovani*, with Pro only influencing the rate of volume decrease, while Ala is involved in controlling the extent of swelling. The authors also suggested that the regulatory volume decrease is mediated by Pro, either through its flow along with the water flow, or associated with activation of a water channel, possibly aquaporin 1 [78].

All functions of amino acids that have been described in TriTryps and mentioned in this review are summarized in Table 3. 

## 5. Concluding Remarks

TriTryps transit during their life cycle through different environments, thus being exposed to variations in their nutritional situations and challenged by a variety of stress conditions. Amino acids are present at different concentrations and ratios in each of the environments and exert different roles in the parasites in the successive life-cycle stages. Amino acids are a group of metabolites with an amazing diversity in their physicochemical properties. In fact, most of physicochemical conditions of any cellular or subcellular microenvironment (such as pH, redox potential, osmolarity) could be created or regulated by using different combinations of amino acids and their intermediate metabolites. In addition to being taken up or synthesized for their role as constituents of proteins, amino acids participate in a myriad of biological processes, such as cell bioenergetics, the regulation of cell cycle, regulation of cell volume, mechanisms of cell survival (autophagy) and death, differentiation, and cell invasion. Even more, in some cases, the regulation of amino acids and their metabolism can subvert the host immune response. The regulation of these activities is dependent on several factors, mainly their uptake, degradation, and biosynthesis. These activities can also be determined by the accumulation of intermediate metabolites, which, in many cases, were shown to be much more than inert entities in the route from precursor to the product of a metabolic pathway. A more detailed knowledge on this intricate metabolic network is necessary to improve our understanding of the biology of these organisms.

## Figures and Tables

**Table 1:** Biochemically-characterized amino acid transport systems and their genes in TriTryps.

Amino Acid	*T. brucei*	*T. cruzi*	*Leishmania* spp.	Gene Name
Proline ^a^	Two systems [38,41,42]	Two systems [54]	Three systems [72]	*Tb*AAT6 [40]
				*Tc*AAP24 [55]
				*Lm*AAP24 [76]
Glutamate		One system [60]	One system [76]	
Glutamine		One system (Damasceno et al., submitted)		
Isoleucine, Leucine and Valine ^b^		One system [64]		
Aspartate		One system [59]		
Arginine ^c^		Two systems [48,49]	One system [73]	*Tb*AAT5-3 [45]
				*Tc*AAAP411/*Tc*AAP3 and *Tc*CAT1.1 [51,53]
				*Ld*AAP3 and *La*AAP3 [75,79,80,81,82]
Ornithine ^d^				*Tb*AAT10-1 and *Tb*AAT2-4 [46]
				*Tc*CAT1.1 [55]
Histidine ^e^		One system [61]		*Tb*AAT2-4 [46]
Methionine	Two systems [43,44]		One system [65,66]	
Lysine ^f^		One system [52]	One system [52]	*Tb*AAT16-1 [45]
				*Tc*AAP7 [52]
				*Lm*AAP7 [52]
Threonine	One system [35]			
Cysteine	One system [36]	One system [63]		
Serine			One system [77]	

^a^ *T. brucei* is auxotrophic for Pro [39], ^b^ TriTryps are auxotrophic for branched-chain amino acids [83,84,85], ^c^ TriTryps are auxotrophic for Arg [85,86], ^d^ *T. brucei* and *T. cruzi* are auxotrophic for Orn [2,87,88,89], ^e^ *T. cruzi* is auxotrophic for His [61,86], ^f^ TriTryps are auxotrophic for Lys [52,90,91].

**Table 2:** Genes annotated as encoding relevant amino acid metabolism enzymes in TriTryps.

Amino Acid	Enzymes	Genes *			Characterization
		*T. brucei*	*T. cruzi*	*L. major*	
Ala	ALAT	Tb927.1.3950	TcCLB.506529.420●	LmjF12.0630	[129,269]
	AlaR	Tb927.5.1280●	TcCLB.509767.90●	LmjF.23.1480	[262]
	TAT	(see aromatic AA)			
Arg	ARG	-	-	LmjF.35.1480	[141]
	ArgK	Tb927.9.6210	TcCLB.482369.29	-	[51,148,150]
Aromatic AA	ASAT/BSAT	Tb927.10.3660	TcCLB.503841.70	LmjF.35.0820	[171,174,228]
	TAT	-	TcCLB.508535.50	LmjF.36.2360	[129]
Asn	ASN	-	TcCLB.504147.110●	LmjF.36.4430●	
Asp	AS	Tb927.7.1110	TcCLB.503899.90●	LmjF.26.0830●	[176]
	ASAT	(see aromatic AA)			
	mASAT	Tb927.11.5090	TcCLB.510945.70	LmjF.24.0370●	[174]
BCAA	BCAT	Tb927.2.4590●		LmjF27.2030●	
	TAT/ASAT	(see aromatic AA)			
Cys	CGS	Tb11.v5.0869●	TcCLB.510741.10●	LmjF14.0460	[215]
	CD	-	-	LmjF.32.2640	[216]
Gln	GLNAT	Tb927.10.11970	TcCLB.503747.10●	LmjF.33.1330●	[129]
	GF6PA	Tb927.7.5560●	TcCLB.510303.200	LmjF.06.0950	[131]; Crispim et al., unpublished data
	CPS	Tb927.5.3800●	TcCLB.508373.10	LmjF.16.0590	[126,127]
	GMPS	Tb927.7.2100	TcCLB.508085.10●	LmjF.22.0110●	[128]
Glu	GDH	Tb927.9.5900●	TcCLB.505843.10	LmjF.15.1010●	[119]
	GS	Tb927.7.4970●	TcCLB.503405.10	LmjF.06.0370	[125,130]
	P5CS	-	TcCLB.509067.70●	LmjF.32.3140●	
	TAT/ASAT	(see aromatic AA)			
Gly	GCC-P	Tb927.7.1910●	TcCLB.510911.50●	LmjF26.0030	[244]
His	HAL	-	TcCLB.506247.220	-	[61]
Lys	-	-	-	-	
Met	AdoMetS	Tb927.6.4840	TcCLB.506945.160●	LmjF.30.3500	[189,191]
	MSR	Tb427.08.550●	TcCLB.510855.10●	LmjF.07.1140●	
Pro	PRODH	Tb927.7.210	TcCLB.506411.30	LmjF.26.1610	[38,95]; Ferraz et al., unpublished data
	PRAC	-	TcCLB.430737.10●	-	
Phe	PAH	-	-	LmjF.28.1280	[229]
Ser	SHMT-L	-	-	LmjF.28.2370	[241]
	SHMT-S	-	TcCLB.510407.90	LmjF.14.1320	[241,243]
	SAT		TcCLB.510879.80	LmjF.34.2850	[210,216]
	CBS	Tb11.02.5400●	TcCLB.508241.140	LmjF17.0250	[210,212]
Thr	TDH	Tb927.6.2790	TcCLB.507923.10●	-	[169]
● Putative genes					

* Accession numbers from reference strains: *T. brucei* TREU927, *T. cruzi* CL Brener Esmeraldo-like (except HAL) and *L. major* strain Friedlin.

**Table 3:** Functions associated to amino acids in TriTryps.

Amino Acid	Function	*T. brucei*	*T. cruzi*	*Leishmania* spp.	References
Ala	Osmotic control				[336,337,338]
Arg	Polyamine biosynthesis				[134,136]
	Establishment of infection				[306]
	Apoptosis protection				[320]
	Energy management				[51,148,149]
Asn	Metacyclogenesis				[278]
Asp	Metacyclogenesis				[172]
	Pyrimidine biosynthesis				[175]
	Mitochondrial ATP synthesis				[93]
Cys	Trypanothione synthesis				[214]
Gln	Pyrimidine biosynthesis				[126,127]
	Hexosamine biosynthesis				Crispim et al., unpublished; [131]
	Metacyclogenesis				Damasceno et al., submitted
Glu	Osmotic control				[336,337,338]
	Metacyclogenesis				[172]
	Mitochondrial ATP synthesis				[93,121]
	Resistance to oxidative stress				[277]
	Resistance to nutritional stress				[277]
Gly	Osmotic control				[336,337,338]
His	Resistance to nutritional stress				[61]
	Autophagy protection				[316]
Leu	Sterol biosynthesis				[165,167]
	Fatty acid biosynthesis				[165]
Met	Polyamine biosynthesis				[173]
	Resistance to oxidative stress				[197,204,205]
Pro	Mitochondrial ATP synthesis				[2,38,39,92,95,96]
	Carbon source				[2,30,38,103]
	Cell differentiation				[101,172]
	Cell invasion				[96,279,337]
	Osmotic control				[337]
	Resistance to oxidative stress				[276]
	Resistance to thermal stress				[276]
	Resistance to nutritional stress				[276]
Ser	Osmotic control				[336,338]
Thr	Carbon source				[169,256]

## Supplementary Materials

The following item is available online at http://www.mdpi.com/2076-0817/7/2/36/s1, **Table S1.** Enzymes mentioned in the text with their respective abbreviations, EC numbers, and references to figures.
